# Temple syndrome in a patient with variably methylated CpGs at the primary *MEG3/DLK1*:IG-DMR and severely hypomethylated CpGs at the secondary *MEG3*:TSS-DMR

**DOI:** 10.1186/s13148-019-0640-2

**Published:** 2019-03-07

**Authors:** Masayo Kagami, Atsuhiro Yanagisawa, Miyuki Ota, Kentaro Matsuoka, Akie Nakamura, Keiko Matsubara, Kazuhiko Nakabayashi, Shuji Takada, Maki Fukami, Tsutomu Ogata

**Affiliations:** 1 Department of Molecular Endocrinology, National Research Institute for Child Health and Development, 2-10-1 Okura, Setagaya-ku, Tokyo, 157-8535 Japan; 2 Department of Pediatrics, Yaizu City Hospital, 1000 Doubara, Yaizu, Shizuoka 425-8505 Japan; 3 Department of Pediatrics, JR Tokyo General Hospital, 2-1-3 Yoyogi, Shibuya-ku, Tokyo, 151-8528 Japan; 4 Department of Pathology, Dokkyo Medical University, Saitama Medical Center, 2-1-50 Minami-Koshigaya, Koshigaya, Saitama 343-8555 Japan; 5 Department of Pediatrics, Hokkaido University Graduate School of Medicine, Kita 15, Nishi 7, Kita-ku, Sapporo, 060-8638 Japan; 6 Department of Maternal-Fetal Biology, National Research Institute for Child Health and Development, 2-10-1 Okura, Setagaya-ku, Tokyo, 157-8535 Japan; 7 Department of Systems BioMedicine, National Research Institute for Child Health and Development, 2-10-1 Okura, Setagaya-ku, Tokyo, 157-8535 Japan; 8 Department of Pediatrics, Hamamatsu University School of Medicine, 1-20-1 Handayama, Higashi-ku, Hamamatsu, Shizuoka 431-3192 Japan

**Keywords:** Temple syndrome, Multilocus imprinting disturbance, Primary DMR, Secondary DMR

## Abstract

**Background:**

The human chromosome 14q32.2 imprinted region harbors the primary *MEG3/DLK1*:IG-differentially methylated region (DMR) and secondary *MEG3*:TSS-DMR. The *MEG3*:TSS-DMR can remain unmethylated only in the presence of unmethylated *MEG3/DLK1*:IG-DMR in somatic tissues, but not in the placenta, because of a hierarchical regulation of the methylation pattern between the two DMRs.

**Methods:**

We performed molecular studies in a 4-year-old Japanese girl with Temple syndrome (TS14).

**Results:**

Pyrosequencing analysis showed extremely low methylation levels of five CpGs at the *MEG3*:TSS-DMR and grossly normal methylation levels of four CpGs at the *MEG3/DLK1*:IG-DMR in leukocytes. HumanMethylation450 BeadChip confirmed marked hypomethylation of the *MEG3*:TSS-DMR and revealed multilocus imprinting disturbance (MLID) including mild hypomethylation of the *H19/IGF2*:IG-DMR and mild hypermethylation of the *GNAS A/B*:TSS-DMR in leukocytes. Bisulfite sequencing showed markedly hypomethylated CpGs at the *MEG3*:TSS-DMR and irregularly and non-differentially methylated CpGs at the *MEG3/DLK1*:IG-DMR in leukocytes and apparently normal methylation patterns of the two DMRs in the placenta. Maternal uniparental disomy 14 and a deletion involving this imprinted region were excluded.

**Conclusions:**

Such a methylation pattern of the *MEG3/DLK1*:IG-DMR has not been reported in patients with TS14. It may be possible that a certain degree of irregular hypomethylation at the *MEG3/DLK1*:IG-DMR has prevented methylation of the *MEG3*:TSS-DMR in somatic tissues and that a hypermethylation type MLID has occurred at the *MEG3/DLK1*:IG-DMR to yield the apparently normal methylation pattern in the placenta.

**Electronic supplementary material:**

The online version of this article (10.1186/s13148-019-0640-2) contains supplementary material, which is available to authorized users.

## Background

The human chromosome 14q32.2 imprinted region harbors a cluster of imprinted genes, including paternally expressed *DLK1* and *RTL1* and maternally expressed *MEG3*, *RTL1as*, *MEG8*, *snoRNAs*, and *microRNAs* [1, 2]. The parental origin dependent expression patterns of these imprinted genes are regulated by the methylation patterns of two differentially methylated regions (DMRs), i.e., the germline-derived primary *MEG3/DLK1*:IG-DMR and the postfertilization-derived secondary *MEG3*:TSS-DMR [3]. Both DMRs are methylated on the paternally inherited allele and unmethylated on the maternally transmitted allele in somatic tissues such as leukocytes and skin fibroblasts. In the placenta, the *MEG3/DLK1*:IG-DMR alone remains as a DMR with the same methylation pattern, and the *MEG3*:TSS-DMR is rather hypomethylated regardless of the parental origin [1, 4]. Consistent with such methylation patterns, the unmethylated *MEG3/DLK1*:IG-DMR and *MEG3*:TSS-DMR of maternal origin function as imprinting control centers in the placenta and somatic tissues, respectively. Furthermore, the *MEG3/DLK1*:IG-DMR acts hierarchically as an upstream regulator for the methylation pattern of the *MEG3*:TSS-DMR in somatic tissues, but not in the placenta [4]. Thus, the *MEG3*:TSS-DMR can stay unmethylated only in the presence of unmethylated *MEG3/DLK1*:IG-DMR in somatic tissues.

Maternal uniparental disomy 14 (UPD(14)mat), microdeletions involving paternally expressed *DLK1* (and *RTL1*), and epimutations (hypomethylations) affecting both DMRs of paternal origin cause a constellation of clinical features including pre- and post-natal growth failure, muscular hypotonia, feeding difficulties, small hands and feet, and precocious puberty [5]. The name “Temple syndrome” (TS14) has been approved for such conditions affecting the 14q32.2 imprinted region [5]. Thus, the diagnosis of TS14 is primarily based on genetic rather than clinical findings, although the precise definition of TS14 has not yet been established. Importantly, there is no single report of an isolated epimutation of the *MEG3/DLK1*:IG-DMR or the *MEG3*:TSS-DMR in somatic tissues, in agreement with the hierarchical interaction between the two DMRs.

TS14 is associated with Silver-Russell syndrome (SRS)-compatible phenotype and Prader-Willi syndrome (PWS)-like hypotonia with variable expressivity and incomplete penetrance, in infancy to early childhood [6, 7]. Indeed, our recent study in 32 patients with molecularly confirmed TS14 has revealed both SRS-compatible phenotype and PWS-like hypotonia in ~ 50% of patients, SRS-compatible phenotype alone in ~ 20% of patients, PWS-like hypotonia alone in ~ 20% of patients, and non-syndromic growth failure in the remaining ~ 10% of patients in infancy to early childhood [8], when their infantile phenotypes were assessed by the presence or absence of clinical features utilized in the Netchine-Harbison scoring system developed for the clinical diagnosis of SRS [9] and by that of clinical features prompting genetic testing for PWS [10]. From late childhood, however, TS14 patients frequently exhibit truncal obesity inconsistent with SRS and gonadotropin-dependent precocious puberty contrastive to central hypogonadotropism in PWS [8]. Thus, the overall phenotype would argue for TS14 being an independent clinical entity.

Recent studies have identified variable degrees of multilocus imprinting disturbances (MLIDs) in a subset of patients with imprinting diseases (IDs) caused by epimutations [11]. Indeed, MLIDs have been detected in several patients with TS14 caused by epimutations, although their clinical features remain within the phenotypic spectrum of TS14 [12]. The underlying mechanism(s) leading to MLIDs is unknown in most patients, while mutations of causative or candidate genes for MLID have been identified in a certain fraction of patients and/or their mothers (for review, see [13]).

Here, we report a TS14 patient with severely hypomethylated *MEG3*:TSS-DMR and considerably methylated *MEG3/DLK1*:IG-DMR, together with partial MLIDs at several DMRs, and discuss the atypical methylation pattern of the *MEG3/DLK1*:IG-DMR.

## Patient and methods

### Case report

**Fig. 1:**
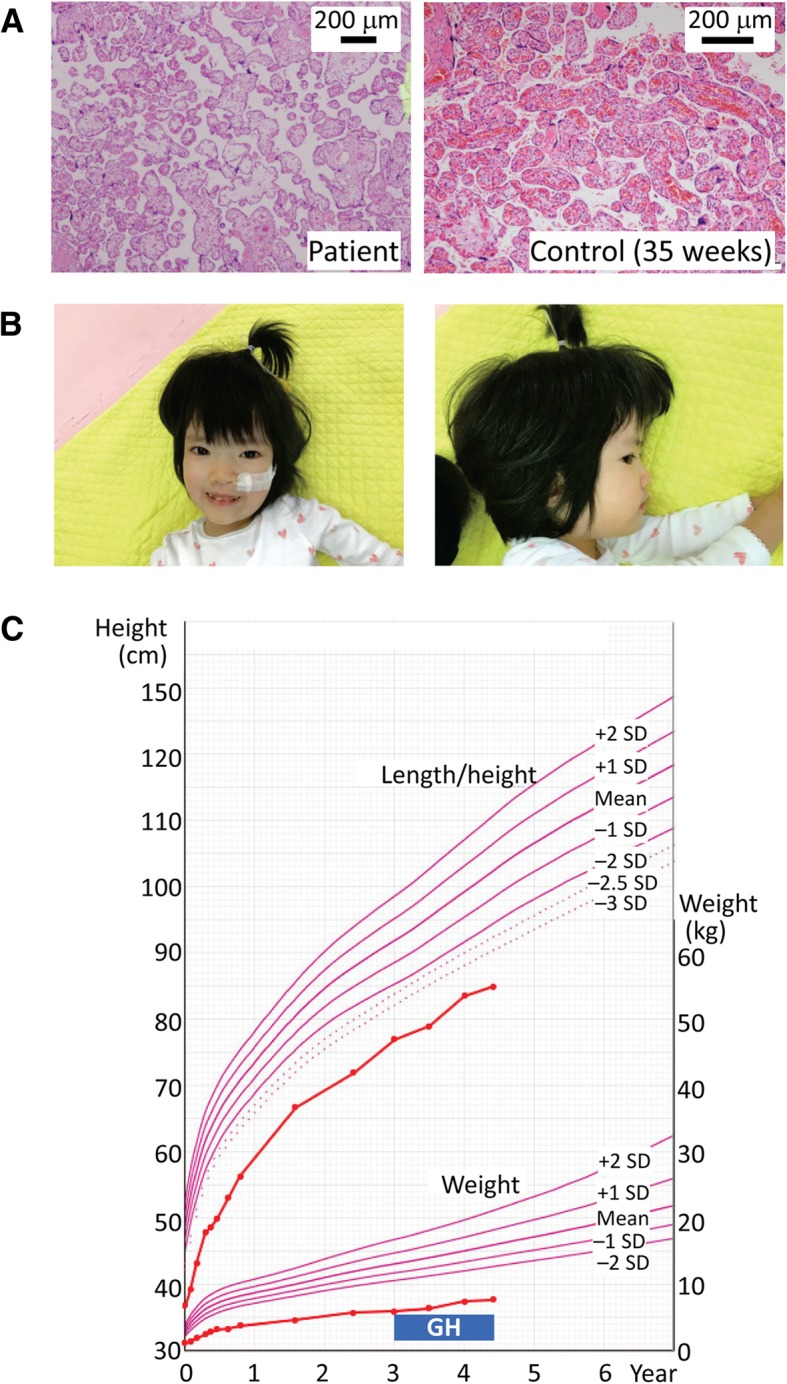
Clinical findings. **a** Histological finding of the placenta. **b** Facial photos at 4 years of age. **c** Growth chart of this patient. She receives growth hormone (GH) therapy (0.23 mg/kg/week) for short stature born small-for-date from 3 years of age

Clinical findings of this Japanese girl are summarized in Table 1. She was conceived naturally to a 35-year-old father and a 38-year-old mother and was delivered by cesarean section at 34 weeks of gestation because of intrauterine growth retardation. The placenta was small and histologically characterized by hypoplastic and edematous villi and chorioamnionitis (Fig. 1a). At birth, her length was 36.8 cm (− 2.9 SD), weight 1.18 kg (− 3.8 SD), and occipitofrontal circumference (OFC) 30.7 cm (− 0.4 SD). Apgar score was seven at 1 min and nine at 5 min. She was admitted to a neonatal intensive care unit for 95 days, to receive tube feeding for feeding difficulty and resulting failure to thrive. Brain magnetic resonance imaging, auditory brainstem response, and echocardiography showed no abnormal findings. Routine laboratory tests were normal, and chromosome analysis revealed a 46,XX karyotype. Physical examination revealed all the six Netchine-Harbison scoring features for SRS (Fig. 1b) [9] and a marked hypotonia characteristic of PWS [10]. While she received growth hormone treatment for short stature born small for gestational age from 3 years of age, she remained small with a relatively large OFC (Fig. 1c). On the last examination at 4 years and 5/12 months of age, her height was 85.0 cm (− 4.4 SD), weight 7.8 kg (− 7.8 SD), and OFC 46.8 cm (− 2.0 SD). She still required tube feeding and showed obvious developmental delay with a developmental quotient of 66.

**Table 1:** Clinical features of this patient

	This patient	TS14 patients (*n* = 32) (Ref. [8])
Genetic causes	Epimutation	UPD(14)mat (*n* = 23) Epimutation (*n* = 6) Paternal deletion (*n* = 3)
Sex (male to female)	Female	18:14
Karyotype	46,XX	…
Pregnancy and delivery		
Gestational age (weeks)	34	39 (30–41) (*n* = 31)
Placental weight g (%)*	195 (47)	74 (56–120) (*n* = 7)
Medically assisted reproduction	–	2/30 (7%)
Paternal age at childbirth (years)	35	33 (22–48) (*n* = 29)
Maternal age at childbirth (years)	38	30 (22–42) (*n* = 31)
Growth		
Birth length-SDS	− 2.9	− 2.1 (− 4.0 to + 1.4) (*n* = 29)
Birth weight-SDS	− 3.8	− 2.7 (− 4.6 to + 3.8) (*n* = 31)
Birth OFC-SDS	− 0.4	−1.2 (− 3.9 to + 1.4) (*n* = 27)
Present age (years to months)	4:5	9.3 (0.7–62) (*n* = 32)
Present height-SDS	− 4.4	− 2.3 (− 8.0 to + 0.2) (*n* = 32)
Present weight-SDS	− 7.8	− 1.5 (− 5.7 to + 4.3) (*n* = 32)
Present OFC-SDS	− 2.0	− 1.8 (− 4.9 to − 0.7) (*n* = 13)
TS14 clinical features		
Pre- and/or post-natal growth failure	+	31/32 (97%)
Obesity	–	…
Muscular hypotonia	+	21/31 (66%)
Small hands	+	29/32 (91%)
Feeding difficulty	+	19/30 (63%)
Early onset of puberty	…	13/17 (76%)
SRS Netchine-Harbison scoring system criteria	6/6	4 (0–6) (*n* = 21)
Birth length and/or weight ≤ – 2 SDS	+	26/31 (84%)
Height at ~ 2 years ≤ − 2 SDS	+	24/26 (93%)
Relative macrocephaly at birth^†^	+	14/27 (52%)‡
Prominent forehead (1–3 years)	+	19/30 (63%)
Body asymmetry	+	7/30 (23%)
Feeding difficulties	+	19/30 (63%)
PWS salient features prompting genetic testing < 6 years		
Hypotonia (with poor suck)	+	21/31 (68%)
Global developmental delay (≥ 2 years)	+	5/26 (19%)
Developmental status		
Age at head control (months)	18	6.5 (3–10) (*n* = 25)
Age at sitting without support (months)	24	10 (6–15) (*n* = 25)
Age at standing with support (years)	4 5/12	…
Intellectual disability	+	2/12 (17%)
Speech delay	+	…
IQ/DQ	DQ = 66 (at 4 5/12 years)	90 (53–114) (*n* = 12)
Neurological and/or emotional problems	–	5/32 (16%)
Other findings		
Joint hypermobility	+	10/30 (33%)
Scoliosis	–	6/32 (19%)
Recurrence otitis media	–	…
Clinodactyly	–	11/28 (39%)

The data of the previously reported 32 patients with TS 14 are shown as the median (range) or frequency. For frequency, the denominators indicate the number of patients examined for the presence or absence of each feature, and the numerators represent the number of patients assessed to be positive for that feature; thus, the differences between the denominators and the numerators denote the number of patients evaluated to be negative for that feature

*OFC* occipitofrontal circumference

*Assessed by the gestational age-matched placental weights [23]

†Birth OFC-SDS ≥ 1.5 above birth length-SDS and/or birth weight-SDS

‡Postnatal relative macrocephaly is found in 38% of patients

### Ethical approval

This study was approved by the Institutional Review Board Committees at National Center for Child Health and Development and was performed after obtaining written informed consent.

### Samples and primers

Genomic DNA (gDNA) was extracted from fresh leukocytes with FlexiGene DNA Kit (Qiagen, Hilden, Germany) and from paraffin-embedded placenta with AllPrep DNA/RNA FFPE Kit (Qiagen). Metaphase spreads were prepared from lymphocytes. Total RNA was obtained from Epstein-Barr virus-transformed lymphoblastoid cell lines with an AllPrep DNA/RNA/miRNA Universal Kit (Qiagen), and cDNA samples were prepared with oligo (dT) primers using Superscript III Reverse Transcriptase (Thermo Fisher Scientific, Waltham, MA, USA) or TaqMan Advanced miRNA Assays (Thermo Fisher Scientific). Primers utilized in this study are listed in Additional file 1: Table S1.

### Methylation analysis

Methylation analysis was performed for 49 CpG sites at nine DMRs, including four CpGs at the *MEG3/DLK1*:IG-DMR and five CpGs at the *MEG3*:TSS-DMR, involved in the development of known IDs, by pyrosequencing with PyroMark Q24 (Qiagen), and for 753 CpG sites at multiple DMRs widely distributed on the genome, including the *MEG3*:TSS-DMR but not the *MEG3/DLK1*:IG-DMR, with HumanMethylation450 BeadChip (Illumina, San Diego, CA, USA), using bisulfite-treated leukocyte gDNA samples. The data from 50 and 11 healthy subjects were utilized as references for pyrosequencing and HumanMethylation450 BeadChip, respectively. In HumanMethylation450 BeadChip analysis, each CpG site was interpreted as abnormally methylated when the |Δβ| was > 3 SD and > 0.05, and each DMR was assessed as abnormally methylated when > 20% of CpGs within the DMR showed abnormal methylation levels, as employed previously [12]. These analyses were not performed for placental gDNA because of a lack of reference data. Furthermore, bisulfite sequencing was carried out for six CpGs at the *MEG3/DLK1*:IG-DMR and seven CpGs at the *MEG3*:TSS-DMR using leukocyte and placental gDNA samples. Of the 49 CpGs examined by pyrosequencing, only four CpGs (CpG14, CpG15, and CpG18 at the *PEG10*:TSS-DMR; and CpG45 at the *GNAS A/B*:TSS-DMR) were included in the list of HumanMethylation450 BeadChip, whereas all nine CpGs at the *MEG3/DLK1*:IG-DMR and *MEG3*:TSS-DMR were analyzed by bisulfite sequencing. The detailed methods have been described previously [4, 6, 14].

### UPD and deletion analyses

We performed microsatellite analysis for seven loci on chromosome 14, genome-wide array comparative genomic hybridization (aCGH) and single-nucleotide polymorphism (SNP) array using SurePrint G3 ISCA CGH + SNP Microarray Kit (catalog number G4890, 4 × 180 K format) (Agilent Technologies, Santa Clara, CA, USA), aCGH using a custom-build dense oligo-microarray for chromosome 14q32.2–q32.3 (Design ID 032112, Agilent Technologies), and fluorescence in situ hybridization (FISH) for the *MEG3/DLK1*:IG-DMR and *MEG3*:TSS-DMR, as reported previously [1, 14].

### Expression analysis

We performed quantitative PCR analysis using TaqMan real-time PCR Assay. cDNA samples were subjected to an ABI PRISM 7000 (Thermo Fisher Scientific) with probe-primer mixtures (catalog assay No: Hs00262142 for *H19*, Hs04188276 for *IGF2*, Hs00171584 for *DLK1*, Hs00292028 for *MEG3*, and Hs00419701 for *MEG8*; assay ID: 477901_mir for *miR134*-5p; and custom assay ID: AJ70MG9 for *GNAS*-*A/B*) (Thermo Fisher Scientific). Data were normalized against *GAPDH* (catalog No: 4326317E), except for *miR134*-5p which were normalized against *miR361*-5p (assay ID: 478056_mir) (Thermo Fisher Scientific).

### Whole exome sequencing

Whole-exome sequencing was carried out for the patient and her parents with SureSelect Human All Exon V5 (Agilent Technologies). Captured libraries were sequenced with a HiSeq 1500 (Illumina) with 101-base pair (bp) paired-end reads, as reported previously [12]. Reads from each sample were trimmed by removing adapters and low-quality bases at ends using cutadapt 1.7.1 and were mapped to the hs37d5 (GRCh37) reference sequence using the BWA 0.7.12. PCR duplicates were removed by Picard 1.83. Multi-sample calling for single-nucleotide and short indel variations was performed by GATK 2.8. Common variants were excluded on the basis of 1000 genomes project data [15] and Human Genetic Variation Database [16].

## Results

### Methylation analysis

**Fig. 2:**
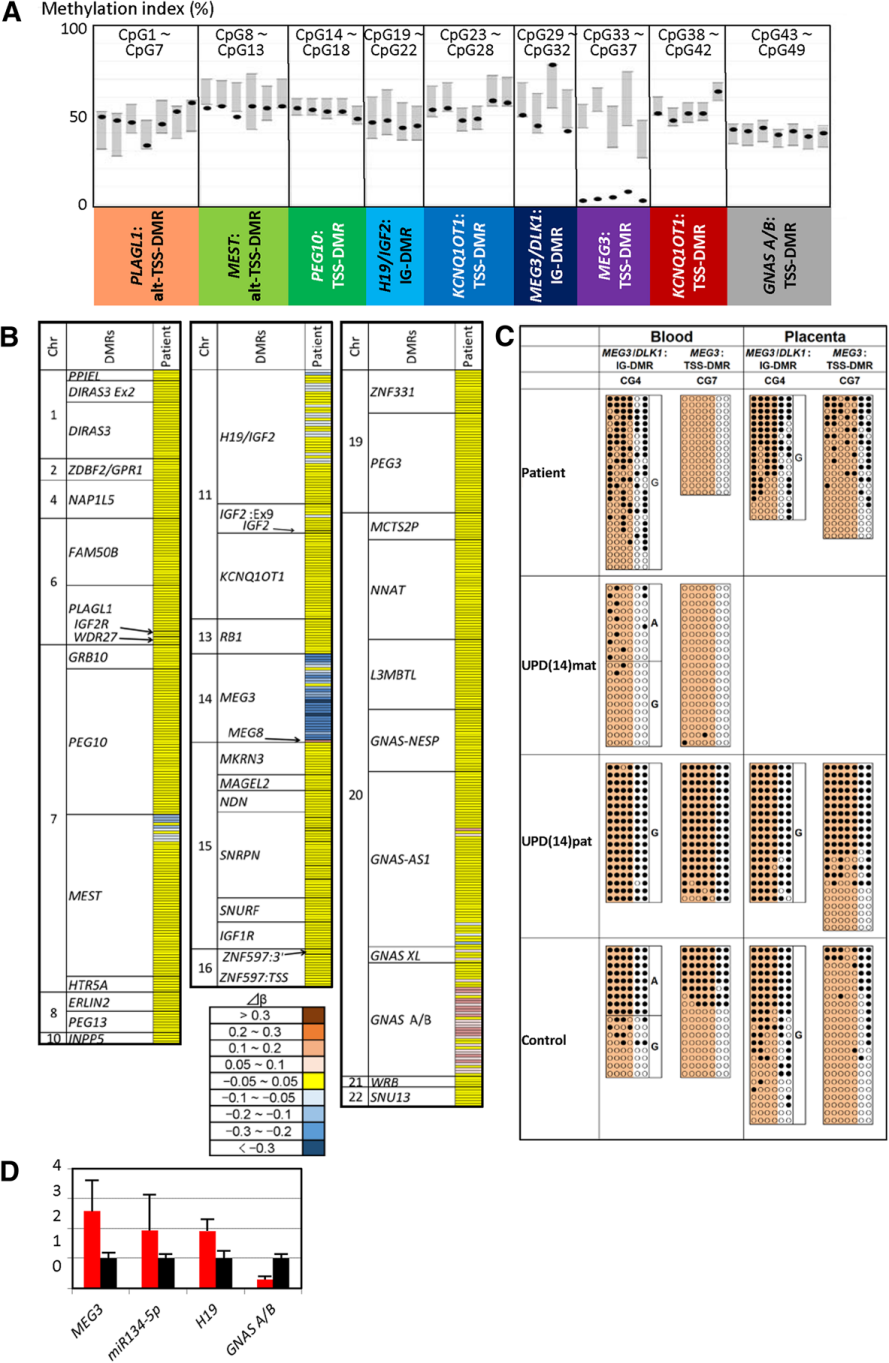
The results of methylation and expression analyses. **a** Methylation indices (MIs, the ratios of methylated CpGs at each CpG site) obtained by pyrosequencing for 49 CpGs. Black circles represent the mean MIs calculated after four times of analyses in this patient, and gray vertical bars indicate the reference ranges (minimum–maximum) obtained from 50 control subjects. **b** Heatmap indicating the Δ*β* values for 753 CpG sites examined by the HumanMethylation450 BeadChip. A single row indicates a single CpG site. The methylation levels of CpG sites are classified into nine categories based on Δ*β* values. For the formal nomenclature of examined DMRs/loci, see Monk et al. [22]. **c** Bisulfite sequencing analysis for the *MEG3/DLK1*:IG-DMR (CG4) and *MEG3*:TSS-DMR (CG7). Each line indicates each clone, and filled and open circles represent methylated and unmethylated cytosines at the CpG dinucleotides, respectively. The four CpGs at the *MEG3/DLK1*:IG-DMR and five CpGs at the *MEG3*:TSS-DMR highlighted in orange have also been examined by pyrosequencing. Since the CG4 region contains a G/A SNP (*rs12437020*), genotyping data for this SNP are also shown; the leukocytes and placental samples are derived from different control subjects as indicated by different genotyping data. **d** Quantitative real-time PCR analysis using immortalized lymphocytes. Shown are relative mRNA expression levels of *MEG3*, *H19*, *GNAS*-A/B, and *miR134*-5p (mean ± SE). The expression studies were performed three times for each sample

Pyrosequencing analysis showed extremely low methylation levels of five CpGs at the *MEG3*:TSS-DMR and variable but grossly normal methylation levels of four CpGs at the *MEG3/DLK1*:IG-DMR with a relatively hypermethylated CpG and a relatively hypomethylated CpG, as well as normal methylation levels of most CpGs at the remaining seven DMRs (Fig. 2a). The methylation level of each CpG was well reproduced by four times of independent analyses with a variation in the methylation index within 2%. HumanMethylation450 BeadChip analysis also revealed marked hypomethylation of nearly all CpGs at the *MEG3*:TSS-DMR accompanied by a hypermethylated CpG at the *MEG8*-DMR [12], together with mild hypomethylation of 30% of CpGs at the *H19/IGF2*:IG-DMR and mild hypermethylation of 64% of CpGs at the *GNAS A/B*:TSS-DMR, in addition to a few of hypo- or hypermethylated CpGs at several DMRs (Fig. 2b) (for details, see Additional file 1: Table S2 and its footnotes). The Beadchip analysis was performed once. For the *H19/IGF2*:IG-DMR, the CpGs around CpG19–22 examined by pyrosequencing were shown to be normally methylated by HumanMethylation450 BeadChip analysis; for the *GNAS A/B*:TSS-DMR, the CpGs around CpG43–49 examined by pyrosequencing were found to be hypermethylated by HumanMethylation450 BeadChip analysis. (Additional file 1: Figure S1). Bisulfite sequencing disclosed markedly hypomethylated CpGs at the *MEG3*:TSS-DMR and rather irregularly (non-differentially) methylated CpGs at the *MEG3/DLK1*:IG-DMR in leukocytes and apparently similar methylation patterns of the *MEG3/DLK1*:IG-DMR and *MEG3*:TSS-DMR between placentas of this patient and a control subject (Fig. 2c).

### UPD and deletion analyses

UPD (14) mat and a deletion involving the 14q32.2 imprinted region were excluded (Additional file 1: Table S3 and Additional file 1: Figure S2).

### Expression analysis

Quantitative PCR analyses showed increased expression of *MEG3* and *miR134*-5p regulated by the *MEG3*:TSS-DMR, increased expression of *H19* controlled by the *H19/IGF2*:IG-DMR, and decreased expression of *GNAS A/B* regulated by the *GNAS A/B*-DMR (Fig. 2d). *IGF2*, *DLK1*, and *MEG8* expression was not detected.

### Whole exome sequencing

No pathogenic variant was identified in causative or candidate genes for MLID, such as *ZFP57*, *NLRP2*, *NLRP7*, *KHDC3L*, *NLRP5*, *TRIM28*, *PADI6*, *OOEP*, *UHRF1*, and *ZAR1* [13], of this patient and the parents. Additionally, because no cSNP was identified in *MEG3*, *miR134*-5p, and *H19*, biparental expression of these genes was not demonstrated.

## Discussion

We identified an epimutated (hypomethylated) *MEG3*:TSS-DMR in leukocyte gDNA of a 4-year-old girl with typical SRS features and PWS-like marked hypotonia. Notably, the *MEG3*:TSS-DMR alone was found to be severely hypomethylated by the different methylation analysis methods, while mildly hypo- or hypermethylated CpGs were also detected in other DMRs by HumanMethylation450 BeadChip analysis. On the basis of the definitive genetic aberration at the 14q32.2 imprinted region and the presence of both SRS-compatible phenotype and PWS-like phenotype, we made the diagnosis of TS14 in this patient, although the diagnosis of SRS with an aberrant 14q32.2 imprinted region would also be acceptable at this age [8].

The *MEG3/DLK1*:IG-DMR showed confounding methylation patterns which were inconsistent with the markedly hypomethylated *MEG3*:TSS-DMR in leukocyte gDNA and obvious placental hypoplasia. Indeed, pyrosequencing indicated variable but grossly normal methylation levels for the four CpGs at the *MEG3/DLK1*:IG-DMR in leukocytes, and bisulfite sequencing showed a considerable degree of irregular (non-differential) methylation pattern in leukocytes and apparently normal methylation pattern in the placenta. Although the methylation levels obtained by pyrosequencing are known to be higher for the *MEG3/DLK1*:IG-DMR than for the *MEG3*:TSS-DMR in patients with TS14 [6, 12], such methylation patterns with grossly normal methylation levels at the *MEG3/DLK1*:IG-DMR have not been reported in patients with TS14. Furthermore, variable methylation levels ranging from an apparently normal level to a severely skewed level at different DMRs of a single imprinted region have been described only for *GNAS*-DMRs in patients with sporadic pseudohypoparathyroidism type Ib [17]. Since the grossly normal methylation levels indicated by pyrosequencing were found to be due to irregular methylation rather than to differential methylation by bisulfite sequencing, this implies that normal methylation levels do not necessarily represent a differential methylation pattern.

The underlying factor(s) leading to the atypical methylation patterns of the *MEG3/DLK1*:IG-DMR remains to be clarified. However, the *MEG3/DLK1*:IG-DMR are more methylated than the *MEG3*:TSS-DMR in leukocytes of patients with TS14, as described above [6, 12]. Furthermore, Kota et al. have reported that the unmethylated *Meg3/Dlk1*:IG-DMR of maternal origin harbors bidirectionally expressed *cis*-acting relatively short (mostly < 500 bp and up to 750 bp) non-coding RNAs (IG-DMR RNA) that exerts enhancer-like functions on the *Meg3* promoter and protects the *Meg3*:TSS-DMR from de novo methylation in mice [18]. Thus, it may be possible that a certain degree of irregular hypomethylation at the *MEG3/DLK1*:IG-DMR, as observed in this patient, can prevent methylation of the *MEG3/DLK1*:IG-DMR in leukocytes (somatic tissues) by producing a reduced but functionally sufficient amount of IG-DMR RNAs. In this case, the methylation pattern of the *MEG3/DLK1*:IG-DMR in the placenta may be explained by assuming that the *MEG3/DLK1*:IG-DMR was once hypomethylated to produce placental hypoplasia and, subsequently, subjected to hypermethylation type MLID in a relatively late developmental stage. However, it may also be possible that methylated clones were preferentially amplified because of the poor quality of gDNA samples extracted from the paraffin-embedded placenta. In addition, this hypermethylation type MLID might have taken place to a lesser degree in leukocytes (somatic tissues) than in the placenta.

MLID was identified in several DMRs of this patient by HumanMethylation450 BeadChip analysis. The MLID may be involved in the phenotypic development of this patient. Indeed, hypomethylation of the *H19/IGF2*:IG-DMR and hypermethylation of the *GNAS A/B*:TSS-DMR are frequently associated with SRS somatic features including compromised body and placental growth [9, 19]. Since quantitative PCR analysis revealed not only increased expression of *MEG3* and *miR134*-5p but also elevated expression of *H19* and decreased expression of *GNAS A/B*, it is likely that the MLID resulted in altered expression of relevant imprinted genes, contributing to the development of SRS phenotype with persistent severe growth failure unresponsive to growth hormone treatment and postnatal relative macrocephaly which is infrequent in TS14 [8]. In addition, since patients with MLIDs are frequently associated with developmental delay [13], MLID may also be relevant to the obvious developmental delay of this patient.

Several matters should also be pointed out with regard to the MLID. First, the MLID remained relatively mild and occurred at several CpGs at each affected DMR in the absence of a mutation in causative or candidate genes for MLID. This implies that the MLID has taken place as an incidental posy-zygotic event. Second, the mild hypomethylation of the *H19/IGF2*:IG-DMR and hypermethylation of the *GNAS A/B*:TSS-DMR were indicated by HumanMethylation450 BeadChip analysis, but not by pyrosequencing analysis, as reported previously [12], although HumanMethylation450 BeadChip analysis was performed just once. In this regard, the number of examined CpGs is much larger in HumanMethylation450 BeadChip analysis than in pyrosequencing analysis, and the methylation level of each CpG is evaluated by the Δβ value in HumanMethylation450 BeadChip analysis and by the comparison with the normal range (within the normal range or not) in pyrosequencing analysis. These factors would explain why HumanMethylation450 BeadChip analysis is more powerful for the detection of abnormal methylation levels than pyrosequencing. Third, MLID occurred not only as a hypomethylation type but also as a hypermethylation type. Such MLIDs with both hypomethylated and hypermethylated DMRs in the absence of a gene mutation have been identified in multiple patients (Additional file 1: Figure S3). Although the examined CpGs and the utilized methylation analysis methods are variable among patients, the data imply that hypomethylation is more prevalent than hypermethylation and that hypomethylation primarily occurs at various primary DMRs whereas hypermethylation primarily takes place at several specific secondary DMRs such as the *ZDBF2*/*GPR1*:IG-DMR, *ZNF597*:TSS-DMR, *MEG8*:Int2-DMR, *GNAS-NESP*:TSS-DMR, and *GNAS A/B*:TSS-DMR. Thus, it might be possible that defective methylation maintenance of primary DMRs occurs incidentally in the post-zygotic period, followed by hypermethylation of specific DMRs that are hypermethylated when adjacent primary DMRs are hypomethylated, as has been reported for the above secondary DMRs [11, 12, 20, 21]. However, this notion remains purely speculative and awaits further investigations.

## Conclusion

We identified a considerably methylated *MEG3/DLK1*:IG-DMR and severely hypomethylated *MEG3*:TSS-DMR in a patient with typical TS14 somatic and placental phenotype. These data will help clarify the hierarchical interaction between the two DMRs in somatic tissues and the biological function of the primary DMR in the placenta.

## Additional file

Additional file 1: **Table S1.** Primers utilized in this study. **Table S2.** Methylation levels (β-values) of each CpG site in leukocyte DNA samples. **Table S3.** The results of micosatellite analysis. **Figure S1.** Methylation analyses of the *H19/IGF2*:IG-DMR, *MEG3*:TSS-DMR, and *GNAS A/B*:TSS-DMR, using leukocyte gDNA samples. **Figure S2.** Lack of UPD (14) mat and microdeletion in this patient. **Figure S3.** Representative data in patients with both hypomethylation-type and hypermethylation-type of MLID in the absence of a mutation in causative or candidate genes for MLID. (PDF 1007 kb)

## Abbreviations

aCGH: Array comparative genomic hybridization
DMR: Differentially methylated region
FISH: Fluorescence in situ hybridization
gDNA: Genomic DNA
ID: Imprinting disorder
MLID: Multilocus imprinting disturbance
OFC: Occipitofrontal circumference
PWS: Prader-Willi syndrome
SNP: Single-nucleotide polymorphism
SRS: Silver-Russell syndrome
TS14: Temple syndrome
UPD(14)mat: Maternal uniparental disomy chromosome 14
