# Supplementary material for: Temple syndrome in a patient with variably methylated CpGs at the primary MEG3/DLK1:IG-DMR and severely hypomethylated CpGs at the secondary MEG3:TSS-DMR
Source: Clin Epigenetics. 2019 Mar 7;11:42. doi: 10.1186/s13148-019-0640-2 (PMC6407230; doi:10.1186/s13148-019-0640-2)
Supplement: Supplementary file 1 — Table S1. Primers utilized in this study. Table S2. Methylation levels (β-values) of each CpG site in leukocyte DNA samples. Table S3. The results of micosatellite analysis. Figure S1. Methylation analyses of the H19/IGF2:IG-DMR, MEG3:TSS-DMR, and GNAS A/B:TSS-DMR, using leukocyte gDNA samples. Figure S2. Lack of UPD (14) mat and microdeletion in this patient. Figure S3. Representative data in patients with both hypomethylation-type and hypermethylation-type of MLID in the absence of a mutation in causative or candidate genes for MLID. (PDF 1007 kb) [file 13148_2019_640_MOESM1_ESM.pdf]

Supplemental Materials

Temple syndrome in a patient with variably methylated CpGs at the primary *MEG3/DLK1*:IG-DMR and severely hypomethylated CpGs at the secondary *MEG3*:TSS-DMR

Table of Contents

Supplemental Table S1

Supplemental Table S2

Supplemental Table S3

Supplemental Figure S1

Supplemental Figure S2

Supplemental Figure S3

Table S1 Primers utilized in this study.

DMR/Locus<br>Chromosome	Forward (5' → 3')<br>Reverse (5' → 3')	Sequence primer<br>Physical position	AT<br>CN
**Pyrosequencing**			
PLAGL1 :alt-TSS-DMR	GGGGTAGTYGTGTTTATAGTTTAG	GGGTAGTYGTGTTTATAGTTTAGT	55
6q24.2	biotin-CCCAAACACCTACCCTAC	chr6: 144329214-144329359	45
PEG10 :TSS-DMR	AGAAATTTGATTGYGTTTTGAGGAGAAT	AGTTTGGYGAAAGGTT	55
7q21.3	biotin-ACCTTTAAAACTTAATTTCCCCATTTAT	chr7: 94285716-94286059	45
MEST :alt-TSS-DMR	GTGTGGTTGGYGGTTTTGGGATTA	TGTTTTTGGGYGAAAATTTTAT	55
7q32.2	biotin-ACACCCCCTCCTCAAATA	chr7: 130132206-130132348	45
H19/IGF2 :IG-DMR	GTTYGGGGGTTTTTGTATAGTATATGGGT	GGTTGTAGTTGTGGAAT	54
11p15.5	biotin-TCCCATAAATATCCTATTCCCAAATAAC	chr11: 2021039-2021317	45
KCNQ1OT1 :TSS-DMR	GGATTTAGAATTAYGATGYGGATTTTA	TTTTGAATTATTATGAGAATTATAG	55
11p15.5	biotin-TCCCATCTACACCTTATAAACA	chr11: 2720333-2720487	45
MEG3/DLK1 :IG-DMR	ATTTGGTATTTGTAGTTTTATGTTAAGATG	AATTGGGTTTGTTAGTAG	54
14q32.2	biotin-AATCAAAACAACTCAAATCCTTTATAAC	chr14: 101275613-101275776	45
MEG3 :TSS-DMR	TTGTGTTTGAATTTATTTTGTTT	GTGTTTGAATTTATTTTGTTT	54
14q32.2	biotin-CCCCAAATTCTATAACAAATTACTCT	chr14: 101292170-101292336	45
SNURF :TSS-DMR	GTTATGGTAGTGGATTAGGGGGATGA	ATAGTGGTGGGGGTT	54
15q11.2	biotin-CCTTCCCCCTACCTCCCA	chr15: 25200788-25200897	45
GNAS A/B :TSS-DMR	GGGATATTTGAGATTTTGAAAGAA	GTTATTTTTTTATTTGGGAGGA	52
20q13.32	biotin-AATACAAAACCTCCCCTACT	chr20: 57463531-57463746	45
**Bisulphite sequencing**			
CG4 (*MEG3/DLK1* :IG-DMR)	TTTTATTATTGAATTGGGTTTGTTAGT		57
14q32.2	ACAATTCCTACTACAAAATTTCAACA	chr14:101275674-101275982	35
CG7 (*MEG3* :TSS-DMR)	TTGTGTTTGAATTTATTTTGTTT		57
14q32.2	CCCCAAATTCTATAACAAATTACT	chr14:101292169-101292336	35
**Microsatellite analysis**			
D14S608	TAAAGGTTTATCCATGCTGTAGC		57
14q12	ACGTGGTACAGGTAGATAAATGG		
D14S588	GCCGAAAGAAAGAAAAAAGG		57
14q23-24.1	CGAATGCATACTTGCTGTTG		
D14S617	TTTTAGGTGGCCACCATCTA		57
14q32.12	CCAGTTTAGGCAACAGAACA		
D14S1006	TTCCACAGGGCAAGCAGTA		57
14q32.2	TTCTGGCAAAACCCAACC		
D14S985	CAGTGTGACCTTAAACAAGTCG		57
14q32.2	CCTGTGGGGTAGATACACGA		
D14S1010	AGATTCTGGACTTGCCAAC		57
14q32.33	GTAGTAGTCAGGGCTTCCTAGAG		
D14S292	CTGTGTGGTGCATCAATG		57
14q32.33	CATGAAGGCAGCCTCA		

AT: annealing temperature (°C); CN: cycle numbers; Y: C or T (pyrimidine); and R: A or G (purine).
Physical positions of the primers are based on the NCBI database (Genome Build 37.1).

Table S2 Methylation levels (β-values) of each CpG site in leukocyte DNA samples

Examined CpG				This patient		Controls (n = 11)			
CHR	MAPINFO	ID	DMR	Average	Δβ	Average	SD	AVG−3SD	AVG+3SD
1	40024971	cg10243676	PPIEL:Ex-DMR	0.6463961	0.006116282	0.6402798	0.0322834	0.5434295	0.7371302
1	40025232	cg11704876	PPIEL:Ex-DMR	0.6538633	-0.016460455	0.6703238	0.0320186	0.574268	0.7663795
1	40025411	cg22862450	PPIEL:Ex-DMR	0.7200186	-0.004467655	0.7244863	0.0238927	0.6528081	0.7961644
1	40025415	cg15057250	PPIEL:Ex-DMR	0.7351566	-0.007631127	0.7427877	0.0279188	0.6590312	0.8265442
1	68512539	cg03641225	DIRAS3:Ex2-DMR	0.399576	0.020916418	0.3786596	0.0184732	0.3232399	0.4340793
1	68512650	cg24871743	DIRAS3:Ex2-DMR	0.6131586	-0.007475645	0.6206342	0.0198708	0.5610217	0.6802468
1	68512777	cg22901840	DIRAS3:Ex2-DMR	0.5302078	0.006836536	0.5233713	0.0149824	0.4784239	0.5683186
1	68512807	cg20149168	DIRAS3:Ex2-DMR	0.6052529	0.009366664	0.5958862	0.0166689	0.5458796	0.6458929
1	68512845	cg13697378	DIRAS3:Ex2-DMR	0.4898882	-0.006860782	0.496749	0.0163391	0.4477318	0.5457662
1	68512928	cg00448707	DIRAS3:Ex2-DMR	0.6027792	-0.014185209	0.6169644	0.019267	0.5591633	0.6747655
1	68512971	cg09118625	DIRAS3:Ex2-DMR	0.5430354	0.005671136	0.5373643	0.018538	0.4817503	0.5929782
1	68513063	cg21808053	DIRAS3:Ex2-DMR	0.4894189	0.015881882	0.473537	0.0086673	0.447535	0.4995391
1	68515788	cg02317907	DIRAS3:TSS-DMR	0.7194357	0.017459991	0.7019757	0.0210726	0.6387579	0.7651936
1	68515872	cg17943391	DIRAS3:TSS-DMR	0.4569397	0.005282218	0.4516575	0.0149272	0.406876	0.496439
1	68515977	cg19114595	DIRAS3:TSS-DMR	0.601905	-0.016282027	0.618187	0.0180048	0.5641727	0.6722014
1	68516080	cg12070746	DIRAS3:TSS-DMR	0.502683	0.003095627	0.4995874	0.0098203	0.4701264	0.5290484
1	68516093	cg11465163	DIRAS3:TSS-DMR	0.6467228	-0.001737482	0.6484603	0.015338	0.6024462	0.6944744
1	68516101	cg16314899	DIRAS3:TSS-DMR	0.6350874	0.027179473	0.6079079	0.0128232	0.5694383	0.6463776
1	68516138	cg25755905	DIRAS3:TSS-DMR	0.4734966	-0.010249691	0.4837463	0.0179231	0.4299771	0.5375154
1	68516272	cg22500004	DIRAS3:TSS-DMR	0.5650154	-0.006290836	0.5713062	0.016037	0.5231953	0.6194171
1	68516279	cg05949203	DIRAS3:TSS-DMR	0.6149407	0.006023009	0.6089177	0.0180083	0.5548929	0.6629425
1	68516374	cg19694923	DIRAS3:TSS-DMR	0.4952858	0.001358973	0.4939268	0.0159916	0.4459519	0.5419017
1	68516453	cg05392265	DIRAS3:TSS-DMR	0.5254716	-0.009500264	0.5349719	0.0182585	0.4801965	0.5897473
1	68516463	cg06191076	DIRAS3:TSS-DMR	0.5464724	-0.003447173	0.5499196	0.0158382	0.5024049	0.5974342
1	68516465	cg16148270	DIRAS3:TSS-DMR	0.6192043	-0.004502409	0.6237067	0.019417	0.5654556	0.6819578
1	68516472	cg16682227	DIRAS3:TSS-DMR	0.4595132	-0.000990536	0.4605037	0.0258981	0.3828093	0.5381981
1	68516518	cg13208159	DIRAS3:TSS-DMR	0.5297913	0.007261009	0.5225303	0.0144948	0.4790458	0.5660148
1	68516627	cg12986021	DIRAS3:TSS-DMR	0.5574834	-0.001036091	0.5585195	0.0190366	0.5014097	0.6156293
1	68516713	cg13099417	DIRAS3:TSS-DMR	0.6133906	-0.005069827	0.6184604	0.0281873	0.5338984	0.7030224
1	68517177	cg27545611	DIRAS3:TSS-DMR	0.4305657	-0.087716618	0.5182823	0.0393991	0.4000849	0.6364797
1	68517205	cg16148134	DIRAS3:TSS-DMR	0.4180758	-0.0802015	0.4982773	0.0517627	0.3429893	0.6535653
1	68517255	cg13605615	DIRAS3:TSS-DMR	0.4714401	-0.044485264	0.5159254	0.0219505	0.450074	0.5817768
1	68517273	cg10038185	DIRAS3:TSS-DMR	0.4670976	-0.091643773	0.5587414	0.0445013	0.4252374	0.6922454
2	207116070	cg25535070	ZBDF2/GOR1:IG-DMR	0.7298689	0.001902427	0.7279665	0.0127206	0.6898047	0.7661282
2	207116401	cg04359324	ZBDF2/GOR1:IG-DMR	0.6740322	0.008387927	0.6656443	0.0232097	0.5960152	0.7352733
2	207118253	cg20471298	ZBDF2/GOR1:IG-DMR	0.6440738	-0.012121945	0.6561957	0.0163515	0.6071413	0.7052502
2	207118288	cg03428109	ZBDF2/GOR1:IG-DMR	0.6876256	-0.022364318	0.7099899	0.0154859	0.6635322	0.7564476
2	207127226	cg25307915	ZBDF2/GOR1:IG-DMR	0.6291717	0.003596127	0.6255756	0.0195859	0.5668179	0.6843332
2	207127364	cg19366042	ZBDF2/GOR1:IG-DMR	0.6858247	0.033625955	0.6521987	0.0265153	0.5726528	0.7317447
2	207129158	cg09448460	ZBDF2/GOR1:IG-DMR	0.4254104	0.008982209	0.4164282	0.0196893	0.3573601	0.4754962
2	207136184	cg10583266	ZBDF2/GOR1:IG-DMR	0.5843978	0.027819618	0.5565782	0.039473	0.4381592	0.6749972
4	89618324	cg18772071	NAP1L5:TSS-DMR	0.5215518	0.028153636	0.4933982	0.0225162	0.4258496	0.5609468
4	89618411	cg17635114	NAP1L5:TSS-DMR	0.5146519	0.011201391	0.5034505	0.0144323	0.4601536	0.5467475
4	89618533	cg05065100	NAP1L5:TSS-DMR	0.5841799	0.022974682	0.5612052	0.0180719	0.5069896	0.6154208
4	89618637	cg12759554	NAP1L5:TSS-DMR	0.5186663	0.024248055	0.4944182	0.0140441	0.4522858	0.5365506
4	89618667	cg01578057	NAP1L5:TSS-DMR	0.5045544	0.022012527	0.4825419	0.0145555	0.4388753	0.5262085
4	89618861	cg27150681	NAP1L5:TSS-DMR	0.6689119	-0.002722255	0.6716342	0.0202628	0.6108456	0.7324227
4	89618982	cg23954636	NAP1L5:TSS-DMR	0.5508028	-0.004103664	0.5549065	0.0175586	0.5022308	0.6075821
4	89619014	cg01174175	NAP1L5:TSS-DMR	0.6112384	0.001863336	0.6093751	0.0151444	0.5639419	0.6548082
4	89619023	cg06617468	NAP1L5:TSS-DMR	0.6104429	-0.003881982	0.6143249	0.0169174	0.5635726	0.6650772
4	89619030	cg18607468	NAP1L5:TSS-DMR	0.634524	-0.017055009	0.651579	0.0172589	0.5998024	0.7033557
4	89619038	cg11300971	NAP1L5:TSS-DMR	0.7065017	0.011754036	0.6947477	0.0238906	0.6230759	0.7664195
4	89619051	cg19151808	NAP1L5:TSS-DMR	0.6410284	0.018680191	0.6223482	0.024458	0.5489741	0.6957223
4	89619085	cg13610072	NAP1L5:TSS-DMR	0.6457302	-0.001787618	0.6475178	0.0201152	0.5871721	0.7078635
4	89619236	cg07539802	NAP1L5:TSS-DMR	0.733314	0.019273773	0.7140402	0.0164729	0.6646217	0.7634588
6	3849095	cg18872973	FAM50B:TSS-DMR	0.7331886	0.001748018	0.7314406	0.0241902	0.6588698	0.8040113
6	3849190	cg17739279	FAM50B:TSS-DMR	0.6515269	0.019210136	0.6323168	0.0247401	0.5580966	0.706537
6	3849235	cg18656763	FAM50B:TSS-DMR	0.4722761	-0.0015432	0.4738193	0.0183307	0.4188273	0.5288113
6	3849272	cg01570885	FAM50B:TSS-DMR	0.5515888	0.020410673	0.5311781	0.0215897	0.466409	0.5959473
6	3849277	cg09821214	FAM50B:TSS-DMR	0.5447869	0.015157227	0.5296297	0.017342	0.4776036	0.5816557
6	3849294	cg07898446	FAM50B:TSS-DMR	0.5016198	0.017822264	0.4837975	0.0278707	0.4001853	0.5674097
6	3849327	cg25195497	FAM50B:TSS-DMR	0.5677111	-0.016113891	0.583825	0.0176713	0.5308111	0.6368389
6	3849331	cg21740964	FAM50B:TSS-DMR	0.5533577	-0.007766745	0.5611244	0.016623	0.5112555	0.6109934
6	3849350	cg13289019	FAM50B:TSS-DMR	0.4442793	0.010209345	0.43407	0.0250694	0.3588618	0.5092781
6	3849381	cg12840312	FAM50B:TSS-DMR	0.5604189	0.028732191	0.5316867	0.0242008	0.4590844	0.604289
6	3849391	cg01905633	FAM50B:TSS-DMR	0.5043511	0.021524218	0.4828269	0.023466	0.4124288	0.553225
6	3849411	cg21177626	FAM50B:TSS-DMR	0.446828	0.016098445	0.4307296	0.0257127	0.3535915	0.5078676
6	3849434	cg03954573	FAM50B:TSS-DMR	0.6231949	0.028108291	0.5950866	0.0188151	0.5386412	0.651532
6	3849442	cg19362611	FAM50B:TSS-DMR	0.6312589	0.031592809	0.5996661	0.0173134	0.547726	0.6516062
6	3849458	cg18197332	FAM50B:TSS-DMR	0.6070232	0.034779355	0.5722438	0.0229757	0.5033167	0.641171
6	3849475	cg04447621	FAM50B:TSS-DMR	0.585716	0.034315727	0.5514003	0.0284077	0.4661772	0.6366233
6	3849536	cg23835083	FAM50B:TSS-DMR	0.6008574	0.026327436	0.57453	0.01909	0.51726	0.6317999
6	3849542	cg18487516	FAM50B:TSS-DMR	0.610714	0.016491009	0.594223	0.0247351	0.5200178	0.6684282
6	3849577	cg12497786	FAM50B:TSS-DMR	0.6233073	-0.004123645	0.6274309	0.0315699	0.5327214	0.7221405

6	3849688	cg03202897	FAM50B:TSS-DMR	0.5598683	0.010932	0.5489363	0.0175936	0.4961556	0.601717
6	3849690	cg21153160	FAM50B:TSS-DMR	0.4895838	-0.007121773	0.4967056	0.0220267	0.4306255	0.5627856
6	3849702	cg25073793	FAM50B:TSS-DMR	0.5393915	0.020011127	0.5193804	0.0191745	0.4618569	0.5769038
6	3849801	cg27445347	FAM50B:TSS-DMR	0.5662047	0.008774973	0.5574297	0.0170852	0.5061741	0.6086854
6	3849818	cg13101072	FAM50B:TSS-DMR	0.559194	0.011788409	0.5474056	0.020275	0.4865806	0.6082306
6	3850106	cg05811173	FAM50B:TSS-DMR	0.5549968	-0.000258991	0.5552558	0.0194478	0.4969125	0.6135991
6	144328421	cg09730369	PLAGL1:alt-TSS-DMR	0.7394822	0.003907718	0.7355745	0.0148647	0.6909803	0.7801686
6	144328482	cg17865602	PLAGL1:alt-TSS-DMR	0.6352171	0.016076964	0.6191401	0.0136796	0.5781014	0.6601789
6	144328917	cg25350411	PLAGL1:alt-TSS-DMR	0.5661373	0.032374	0.5337633	0.0122595	0.4969849	0.5705417
6	144329052	cg07077459	PLAGL1:alt-TSS-DMR	0.6186677	0.031083391	0.5875843	0.01996	0.5277043	0.6474643
6	144329172	cg22378065	PLAGL1:alt-TSS-DMR	0.5941941	0.016251518	0.5779426	0.01273	0.5397525	0.6161326
6	144329331	cg10007452	PLAGL1:alt-TSS-DMR	0.5086828	0.001184255	0.5074985	0.0135831	0.4667491	0.548248
6	144329382	cg22352234	PLAGL1:alt-TSS-DMR	0.593805	0.0128584	0.5809466	0.0142237	0.5382754	0.6236178
6	144329473	cg00702231	PLAGL1:alt-TSS-DMR	0.6592978	0.029718673	0.6295791	0.0161537	0.5811179	0.6780404
6	144329485	cg12757684	PLAGL1:alt-TSS-DMR	0.5235893	0.014840791	0.5087485	0.0190286	0.4516628	0.5658342
6	144329672	cg21526238	PLAGL1:alt-TSS-DMR	0.6077184	0.026454809	0.5812636	0.0193141	0.5233213	0.6392058
6	144329732	cg21952820	PLAGL1:alt-TSS-DMR	0.5422608	-0.037688564	0.5799494	0.0611266	0.3965694	0.7633293
6	144329766	cg05326984	PLAGL1:alt-TSS-DMR	0.5669835	0.021838536	0.545145	0.0250407	0.4700228	0.6202671
6	144329780	cg08263357	PLAGL1:alt-TSS-DMR	0.6107276	0.015437	0.5952906	0.0188196	0.5388318	0.6517494
6	144329789	cg11532302	PLAGL1:alt-TSS-DMR	0.5710708	0.010836418	0.5602344	0.0274667	0.4778344	0.6426344
6	144329802	cg27216384	PLAGL1:alt-TSS-DMR	0.5213553	0.021509382	0.4998459	0.0169639	0.4489542	0.5507376
6	144329829	cg17895149	PLAGL1:alt-TSS-DMR	0.5910562	0.038119991	0.5529362	0.0134084	0.5127111	0.5931613
6	144329887	cg23460430	PLAGL1:alt-TSS-DMR	0.563308	-0.055400018	0.618708	0.0191324	0.5613107	0.6761053
6	160426951	cg12625139	IGF2R:Int2-DMR	0.6959423	0.002097764	0.6938445	0.0341083	0.5915196	0.7961694
6	160427501	cg08350488	IGF2R:Int2-DMR	0.614598	0.038939227	0.5756588	0.0737334	0.3544586	0.7968589
6	170054730	cg19089141	WDR27:Int13-DMR	0.6130894	-0.100435045	0.7135244	0.202382	0.1063783	1.3206706
6	170055155	cg11938672	WDR27:Int13-DMR	0.642094	-0.0065075	0.6486015	0.0263765	0.5694719	0.7277311
6	170055332	cg18322025	WDR27:Int13-DMR	0.5442767	-0.009850864	0.5541276	0.0310518	0.4609723	0.6472828
7	50849168	cg03104936	GRB10:alt-TSS-DMR	0.6359077	0.055909527	0.5799982	0.0313844	0.4858449	0.6741515
7	50849285	cg05998930	GRB10:alt-TSS-DMR	0.7318674	0.046952482	0.6849149	0.0304367	0.5936049	0.7762249
7	50849415	cg25915982	GRB10:alt-TSS-DMR	0.7149341	0.0148345	0.7000996	0.0312981	0.6062053	0.7939939
7	50849639	cg09150232	GRB10:alt-TSS-DMR	0.7885777	0.021198209	0.7673795	0.0215411	0.7027561	0.8320028
7	50849723	cg16349612	GRB10:alt-TSS-DMR	0.6465362	0.010505164	0.636031	0.0213977	0.571838	0.700224
7	50849805	cg23742152	GRB10:alt-TSS-DMR	0.5468902	0.007146173	0.539744	0.0312525	0.4459864	0.6335016
7	50849931	cg08835688	GRB10:alt-TSS-DMR	0.5756932	0.035072109	0.5406211	0.0153459	0.4945835	0.5866587
7	50850564	cg12903171	GRB10:alt-TSS-DMR	0.5062315	0.027996155	0.4782353	0.0145879	0.4344717	0.521999
7	50850870	cg24977055	GRB10:alt-TSS-DMR	0.5591252	0.017042164	0.542083	0.0147746	0.4977593	0.5864067
7	94285642	cg19107595	PEG10:TSS-DMR	0.362795	0.034567927	0.3282271	0.0115291	0.2936397	0.3628145
7	94285672	cg19924104	PEG10:TSS-DMR	0.4427561	0.007551627	0.4352045	0.0207429	0.3729758	0.4974331
7	94285685	cg11589966	PEG10:TSS-DMR	0.5267744	0.009578164	0.5171962	0.0133658	0.477099	0.5572935
7	94285712	cg27435646	PEG10:TSS-DMR	0.5453156	-0.005633409	0.550949	0.0135991	0.5101516	0.5917464
7	94285745	cg01566785	PEG10:TSS-DMR	0.5284543	0.007642209	0.5208121	0.019414	0.4625701	0.579054
7	94285759	cg05819130	PEG10:TSS-DMR	0.5856032	0.003073618	0.5825296	0.0194487	0.5241835	0.6408756
7	94285768	cg10105290	PEG10:TSS-DMR	0.5737557	0.0012716	0.5724841	0.0178663	0.5188853	0.6260829
7	94285777	cg19079047	PEG10:TSS-DMR	0.4552785	0.012569218	0.4427093	0.0147354	0.398503	0.4869155
7	94285784	cg02965180	PEG10:TSS-DMR	0.5865396	0.0197351	0.5668045	0.016331	0.5178116	0.6157974
7	94285814	cg05277165	PEG10:TSS-DMR	0.5201546	0.009449236	0.5107054	0.0157966	0.4633155	0.5580952
7	94285873	cg13660372	PEG10:TSS-DMR	0.4961601	0.011283855	0.4848762	0.0123159	0.4479286	0.5218239
7	94285887	cg06674062	PEG10:TSS-DMR	0.5028126	0.025077809	0.4777348	0.0137574	0.4364626	0.5190069
7	94285902	cg21778340	PEG10:TSS-DMR	0.5554951	0.030653991	0.5248411	0.02228	0.458001	0.5916812
7	94285911	cg07921535	PEG10:TSS-DMR	0.3923048	0.054664555	0.3376402	0.0290711	0.2504269	0.4248536
7	94285942	cg09910998	PEG10:TSS-DMR	0.5405343	0.016825582	0.5237087	0.0161775	0.4751763	0.5722411
7	94285951	cg27393730	PEG10:TSS-DMR	0.5761732	0.014384691	0.5617885	0.0188477	0.5052455	0.6183315
7	94285960	cg08291000	PEG10:TSS-DMR	0.5474182	0.021307036	0.5261112	0.0099197	0.496352	0.5558703
7	94285993	cg27230044	PEG10:TSS-DMR	0.5488794	0.026170245	0.5227092	0.0153118	0.4767738	0.5686445
7	94286086	cg24885794	PEG10:TSS-DMR	0.5162455	0.032484609	0.4837609	0.0104932	0.4522813	0.5152405
7	94286110	cg26997085	PEG10:TSS-DMR	0.5397088	0.035631382	0.5040774	0.01532	0.4581175	0.5500373
7	94286131	cg22331138	PEG10:TSS-DMR	0.6262677	0.011954582	0.6143131	0.0213579	0.5502395	0.6783867
7	94286208	cg16492735	PEG10:TSS-DMR	0.6218741	0.0185719	0.6033022	0.0139623	0.5614154	0.645189
7	94286219	cg09512080	PEG10:TSS-DMR	0.5922224	0.019815518	0.5724069	0.0121279	0.5360231	0.6087906
7	94286232	cg00906934	PEG10:TSS-DMR	0.5680797	0.025427209	0.5426525	0.0125315	0.505058	0.5802469
7	94286243	cg26503018	PEG10:TSS-DMR	0.5958731	0.016595709	0.5792774	0.0100008	0.5492749	0.6092799
7	94286261	cg27120649	PEG10:TSS-DMR	0.4402453	0.0169546	0.4232907	0.0197382	0.3640762	0.4825052
7	94286263	cg21771834	PEG10:TSS-DMR	0.5904964	0.019434491	0.5710619	0.0102195	0.5404035	0.6017203
7	94286267	cg27001184	PEG10:TSS-DMR	0.6186727	0.007635655	0.611037	0.0113595	0.5769586	0.6451155
7	94286294	cg23342787	PEG10:TSS-DMR	0.6163947	-0.000718227	0.6171129	0.0108863	0.5844541	0.6497717
7	94286304	cg14873490	PEG10:TSS-DMR	0.531641	0.017850827	0.5137902	0.0192457	0.4560531	0.5715273
7	94286343	cg03384175	PEG10:TSS-DMR	0.6172147	0.024650773	0.5925639	0.0152511	0.5468107	0.6383171
7	94286351	cg11985632	PEG10:TSS-DMR	0.6347302	0.021760682	0.6129695	0.0157911	0.5655963	0.6603428
7	94286362	cg13797824	PEG10:TSS-DMR	0.6033916	0.013466991	0.5899246	0.0119479	0.554081	0.6257682
7	94286420	cg11175683	PEG10:TSS-DMR	0.6110287	0.021448327	0.5895804	0.0115925	0.5548029	0.6243578
7	94286431	cg04521244	PEG10:TSS-DMR	0.6070902	0.015907064	0.5911831	0.010265	0.5603882	0.621978
7	94286473	cg11562309	PEG10:TSS-DMR	0.5803903	0.019921727	0.5604686	0.0129854	0.5215125	0.5994246
7	94286484	cg18898992	PEG10:TSS-DMR	0.605432	0.017242891	0.5881891	0.0136744	0.5471659	0.6292124
7	94286511	cg05096321	PEG10:TSS-DMR	0.5509483	0.015423518	0.5355248	0.0165995	0.4857264	0.5853232
7	94286521	cg19663555	PEG10:TSS-DMR	0.5958363	0.014827864	0.5810084	0.010011	0.5509754	0.6110414
7	94286526	cg06695761	PEG10:TSS-DMR	0.5831138	0.010715627	0.5723982	0.0073011	0.5504948	0.5943015
7	94286559	cg21853542	PEG10:TSS-DMR	0.6235989	0.013820064	0.6097788	0.0119525	0.5739213	0.6456363
7	94286632	cg04634483	PEG10:TSS-DMR	0.5775709	0.018803291	0.5587676	0.009282	0.5309217	0.5866135

7	94286650	cg09890341	PEG10:TSS-DMR	0.5942821	0.014688827	0.5795933	0.0170116	0.5285585	0.630628
7	94286669	cg20041873	PEG10:TSS-DMR	0.5839181	0.002074964	0.5818431	0.017025	0.5307682	0.632918
7	94286753	cg04303139	PEG10:TSS-DMR	0.5285588	0.013869873	0.5146889	0.0098871	0.4850275	0.5443503
7	94286760	cg03436478	PEG10:TSS-DMR	0.5881773	0.026444282	0.561733	0.0133692	0.5216254	0.6018407
7	94286793	cg05509218	PEG10:TSS-DMR	0.6261457	0.024235209	0.6019105	0.0148701	0.5573003	0.6465206
7	94286834	cg22924867	PEG10:TSS-DMR	0.5943012	0.042579055	0.5517221	0.0172835	0.4998715	0.6035728
7	94286936	cg01169624	PEG10:TSS-DMR	0.6458765	0.024676027	0.6212005	0.0200812	0.5609569	0.6814441
7	94286953	cg03682823	PEG10:TSS-DMR	0.5603937	0.005729373	0.5546643	0.0140212	0.5126008	0.5967279
7	94286955	cg18139769	PEG10:TSS-DMR	0.5635884	0.011592418	0.551996	0.0143464	0.5089568	0.5950352
7	94287186	cg12397924	PEG10:TSS-DMR	0.4133907	-0.016873636	0.4302643	0.0210058	0.367247	0.4932816
7	94287211	cg15696408	PEG10:TSS-DMR	0.305298	-0.003061755	0.3083598	0.0216392	0.2434422	0.3732774
7	94287242	cg22820921	PEG10:TSS-DMR	0.4344575	0.010509373	0.4239481	0.0197657	0.3646509	0.4832454
7	130130122	cg10473158	MEST:alt-TSS-DMR	0.57685	-0.132045855	0.7088959	0.0410835	0.5856455	0.8321462
7	130130155	cg05222671	MEST:alt-TSS-DMR	0.6367831	-0.119301073	0.7560842	0.0272988	0.6741876	0.8379807
7	130130187	cg10767216	MEST:alt-TSS-DMR	0.5510967	-0.108760918	0.6598576	0.0341909	0.5572849	0.7624303
7	130130189	cg12070022	MEST:alt-TSS-DMR	0.5161672	-0.109379682	0.6255469	0.0383191	0.5105895	0.7405043
7	130130203	cg15068552	MEST:alt-TSS-DMR	0.6595053	-0.111967727	0.771473	0.0370496	0.6603243	0.8826218
7	130130288	cg17347253	MEST:alt-TSS-DMR	0.536502	-0.095206027	0.631708	0.0304954	0.5402218	0.7231942
7	130130320	cg02756856	MEST:alt-TSS-DMR	0.6324094	-0.079527873	0.7119373	0.0274938	0.6294557	0.7944188
7	130130383	cg26275543	MEST:alt-TSS-DMR	0.5091245	-0.085928818	0.5950533	0.0171159	0.5437057	0.6464009
7	130130478	cg20826277	MEST:alt-TSS-DMR	0.5034333	-0.057544691	0.560978	0.0176395	0.5080594	0.6138966
7	130130481	cg14584935	MEST:alt-TSS-DMR	0.4930146	-0.061821182	0.5548358	0.0183137	0.4998947	0.6097769
7	130130588	cg24941047	MEST:alt-TSS-DMR	0.7534193	-0.012524627	0.7659439	0.0137109	0.7248112	0.8070767
7	130130596	cg07427065	MEST:alt-TSS-DMR	0.5347213	0.006235064	0.5284862	0.0082663	0.5036874	0.553285
7	130130740	cg02501418	MEST:alt-TSS-DMR	0.6446663	0.026542391	0.6181239	0.0121184	0.5817686	0.6544792
7	130130747	cg08229366	MEST:alt-TSS-DMR	0.6269354	-0.000248273	0.6271837	0.0198666	0.5675839	0.6867834
7	130130918	cg27417677	MEST:alt-TSS-DMR	0.6287177	0.0208814	0.6078363	0.0139298	0.5660468	0.6496258
7	130130995	cg09003373	MEST:alt-TSS-DMR	0.4849824	0.025045673	0.4599367	0.015875	0.4123117	0.5075617
7	130131085	cg14088957	MEST:alt-TSS-DMR	0.4858701	0.022291755	0.4635783	0.0123432	0.4265487	0.500608
7	130131136	cg06100421	MEST:alt-TSS-DMR	0.5294976	0.013146491	0.5163511	0.0175034	0.463841	0.5688612
7	130131138	cg20297423	MEST:alt-TSS-DMR	0.5251802	0.020998355	0.5041818	0.0197136	0.445041	0.5633227
7	130131146	cg05369791	MEST:alt-TSS-DMR	0.4971389	0.011456345	0.4856826	0.0187621	0.4293963	0.5419688
7	130131189	cg08734637	MEST:alt-TSS-DMR	0.5222807	0.006368009	0.5159127	0.0158605	0.4683312	0.5634942
7	130131258	cg13917504	MEST:alt-TSS-DMR	0.5657064	-0.001927864	0.5676343	0.0106654	0.535638	0.5996305
7	130131268	cg25407198	MEST:alt-TSS-DMR	0.5894413	0.011248191	0.5781931	0.0145336	0.5345922	0.621794
7	130131359	cg21667116	MEST:alt-TSS-DMR	0.5385171	0.023487845	0.5150293	0.0162557	0.4662622	0.5637963
7	130131367	cg23156962	MEST:alt-TSS-DMR	0.5016174	0.029781745	0.4718357	0.0116016	0.4370309	0.5066404
7	130131480	cg17079325	MEST:alt-TSS-DMR	0.6118093	-0.003597818	0.6154071	0.0146292	0.5715196	0.6592946
7	130131484	cg04344875	MEST:alt-TSS-DMR	0.64876	-1.64364E-05	0.6487764	0.0111322	0.6153798	0.682173
7	130131566	cg18934293	MEST:alt-TSS-DMR	0.5979592	0.030568209	0.567391	0.0242482	0.4946463	0.6401357
7	130131633	cg00286878	MEST:alt-TSS-DMR	0.6119355	-0.020664964	0.6326005	0.0150305	0.587509	0.677692
7	130131676	cg12347392	MEST:alt-TSS-DMR	0.6101874	0.044284509	0.5659029	0.0210643	0.5027101	0.6290957
7	130131691	cg04786207	MEST:alt-TSS-DMR	0.6567308	0.047606827	0.609124	0.0178044	0.5557108	0.6625371
7	130131709	cg19386484	MEST:alt-TSS-DMR	0.5648971	0.0208354	0.5440617	0.031653	0.4491028	0.6390206
7	130131730	cg26708559	MEST:alt-TSS-DMR	0.5829789	0.009759782	0.5732191	0.0147574	0.528947	0.6174912
7	130131797	cg22705386	MEST:alt-TSS-DMR	0.6404876	0.025301391	0.6151862	0.0137414	0.573962	0.6564104
7	130131826	cg06212135	MEST:alt-TSS-DMR	0.6721561	0.042473727	0.6296824	0.0207071	0.5675612	0.6918035
7	130131829	cg10249538	MEST:alt-TSS-DMR	0.6657158	0.032621118	0.6330947	0.0212147	0.5694505	0.6967389
7	130131869	cg16823958	MEST:alt-TSS-DMR	0.6606497	0.016136455	0.6445132	0.0176157	0.5916662	0.6973603
7	130131885	cg27338480	MEST:alt-TSS-DMR	0.632674	0.020674764	0.6119992	0.0141016	0.5696943	0.6543042
7	130131887	cg09080913	MEST:alt-TSS-DMR	0.6846409	0.023279655	0.6613612	0.0146227	0.6174931	0.7052294
7	130131905	cg13104298	MEST:alt-TSS-DMR	0.6703508	0.020402736	0.6499481	0.0149722	0.6050314	0.6948647
7	130131916	cg07315018	MEST:alt-TSS-DMR	0.5933333	0.015946009	0.5773873	0.0165022	0.5278807	0.6268939
7	130131921	cg21629528	MEST:alt-TSS-DMR	0.4932344	0.017770391	0.475464	0.0187457	0.4192268	0.5317012
7	130131923	cg20050761	MEST:alt-TSS-DMR	0.5382927	0.0135576	0.5247351	0.0146206	0.4808732	0.568597
7	130131931	cg07870293	MEST:alt-TSS-DMR	0.5625581	0.011995236	0.5505629	0.0171908	0.4989904	0.6021353
7	130132161	cg05556276	MEST:alt-TSS-DMR	0.5841059	0.0091873	0.5749186	0.0149471	0.5300772	0.61976
7	130132199	cg17580798	MEST:alt-TSS-DMR	0.607981	0.015612382	0.5923686	0.0228998	0.5236692	0.661068
7	130132259	cg05862114	MEST:alt-TSS-DMR	0.6050782	0.026165827	0.5789124	0.0222383	0.5121976	0.6456271
7	130132265	cg01784351	MEST:alt-TSS-DMR	0.555298	0.025725255	0.5295727	0.0172033	0.4779629	0.5811826
7	130132286	cg27589003	MEST:alt-TSS-DMR	0.4931106	0.030997936	0.4621127	0.014417	0.4188618	0.5053636
7	130132298	cg19344806	MEST:alt-TSS-DMR	0.6147044	0.015102764	0.5996016	0.015877	0.5519705	0.6472328
7	130132305	cg23714917	MEST:alt-TSS-DMR	0.6496372	0.008643564	0.6409936	0.0141451	0.5985583	0.6834289
7	130132319	cg21200654	MEST:alt-TSS-DMR	0.6653253	0.0139422	0.6513831	0.0113393	0.6173651	0.6854011
7	130132360	cg25519926	MEST:alt-TSS-DMR	0.6306797	0.010234691	0.620445	0.0096305	0.5915535	0.6493365
7	130132413	cg09462536	MEST:alt-TSS-DMR	0.4503479	0.036428264	0.4139196	0.0128476	0.3753769	0.4524624
7	130132419	cg22592140	MEST:alt-TSS-DMR	0.5406736	0.034157773	0.5065158	0.0127899	0.4681461	0.5448855
7	130132422	cg03588221	MEST:alt-TSS-DMR	0.6209381	0.004820273	0.6161178	0.0076756	0.5930909	0.6391447
7	130132453	cg13986840	MEST:alt-TSS-DMR	0.632702	0.0447466	0.5879554	0.018983	0.5310063	0.6449045
7	130132727	cg23312013	MEST:alt-TSS-DMR	0.5799993	0.015896564	0.5641027	0.0101632	0.5336132	0.5945922
7	130132790	cg14952237	MEST:alt-TSS-DMR	0.7157246	0.003903109	0.7118215	0.0138401	0.6703012	0.7533417
7	130133110	cg05260959	MEST:alt-TSS-DMR	0.6494975	0.001725664	0.6477718	0.0135449	0.6071372	0.6884064
7	154862770	cg25780543	HTR5A:TSS-DMR	0.3234864	-0.018667082	0.3421535	0.0143485	0.2991081	0.3851989
7	154862969	cg00852398	HTR5A:TSS-DMR	0.5264416	0.004998027	0.5214436	0.0151973	0.4758516	0.5670355
7	154863176	cg25058023	HTR5A:TSS-DMR	0.4971958	-0.007824882	0.5050207	0.0400645	0.3848271	0.6252142
7	154863244	cg03024742	HTR5A:TSS-DMR	0.631358	0.008346073	0.6230119	0.0215499	0.5583622	0.6876617
7	154863338	cg10538202	HTR5A:TSS-DMR	0.6188664	-0.032085318	0.6509517	0.0286244	0.5650786	0.7368249
7	154863381	cg09623773	HTR5A:TSS-DMR	0.7422143	0.026839273	0.715375	0.0435595	0.5846966	0.8460534
8	37605359	cg11496432	ERLIN2:Int6-DMR	0.6400954	-0.002122555	0.642218	0.0207909	0.5798454	0.7045905

8	37605517	cg13346869	ERLIN2:Int6-DMR	0.5277536	-0.001362773	0.5291164	0.0579607	0.3552342	0.7029986
8	37605552	cg05020125	ERLIN2:Int6-DMR	0.4255379	-0.010119673	0.4356576	0.0203238	0.3746861	0.4966291
8	37605717	cg21505509	ERLIN2:Int6-DMR	0.5833045	0.010880973	0.5724235	0.0299914	0.4824493	0.6623977
8	37605783	cg08247852	ERLIN2:Int6-DMR	0.557588	-0.003205409	0.5607934	0.0257449	0.4835588	0.638028
8	37605936	cg09188212	ERLIN2:Int6-DMR	0.7827887	0.0297993	0.7529894	0.015632	0.7060935	0.7998853
8	37605978	cg00450319	ERLIN2:Int6-DMR	0.6741515	0.027495409	0.6466561	0.0171669	0.5951555	0.6981567
8	141108607	cg21926091	PEG13:TSS-DMR	0.7150186	0.034073009	0.6809456	0.015873	0.6333266	0.7285646
8	141108998	cg22502459	PEG13:TSS-DMR	0.6687685	0.019329664	0.6494388	0.0157112	0.6023052	0.6965725
8	141109051	cg13713522	PEG13:TSS-DMR	0.7230406	0.019897336	0.7031433	0.0298223	0.6136764	0.7926102
8	141109731	cg07878171	PEG13:TSS-DMR	0.6295763	-0.006849436	0.6364257	0.0197565	0.5771563	0.6956951
8	141110260	cg03803002	PEG13:TSS-DMR	0.6235736	0.009851173	0.6137224	0.0198378	0.554209	0.6732359
8	141110747	cg00155882	PEG13:TSS-DMR	0.5165862	0.003801455	0.5127847	0.0180115	0.4587502	0.5668192
8	141110900	cg22807877	PEG13:TSS-DMR	0.5195783	0.010317691	0.5092606	0.016337	0.4602495	0.5582717
8	141111080	cg08626916	PEG13:TSS-DMR	0.6167625	0.0350798	0.5816827	0.021194	0.5181006	0.6452648
10	121578137	cg02722214	INPP5F:Int2-DMR	0.6320639	-0.025751227	0.6578151	0.0220931	0.5915359	0.7240944
10	121578384	cg20365618	INPP5F:Int2-DMR	0.5828042	-0.0134572	0.5962614	0.0149729	0.5513427	0.6411801
10	121578561	cg02857557	INPP5F:Int2-DMR	0.6941196	-0.015482464	0.7096021	0.0263992	0.6304045	0.7887996
10	121578639	cg25045785	INPP5F:Int2-DMR	0.6702387	-0.015591991	0.6858307	0.0253742	0.609708	0.7619534
11	2019079	cg11753499	H19/IGF2:IG-DMR	0.5569189	-0.065135636	0.6220545	0.010614	0.5902125	0.6538966
11	2019090	cg13210239	H19/IGF2:IG-DMR	0.4273687	-0.109816782	0.5371855	0.0339647	0.4352914	0.6390795
11	2019116	cg17985533	H19/IGF2:IG-DMR	0.5761077	-0.066251518	0.6423592	0.03041	0.5511291	0.7335893
11	2019129	cg15922305	H19/IGF2:IG-DMR	0.5619968	-0.066246518	0.6282433	0.0363891	0.5190759	0.7374107
11	2019167	cg25289899	H19/IGF2:IG-DMR	0.3703955	-0.144255682	0.5146512	0.0730972	0.2953596	0.7339428
11	2019436	cg09701145	H19/IGF2:IG-DMR	0.477055	-0.099087873	0.5761429	0.0251361	0.5007347	0.651551
11	2019452	cg25437674	H19/IGF2:IG-DMR	0.4161764	-0.0958167	0.5119931	0.0102426	0.4812652	0.542721
11	2019568	cg17769238	H19/IGF2:IG-DMR	0.5243881	-0.064799155	0.5891873	0.0106183	0.5573324	0.6210422
11	2019587	cg04975775	H19/IGF2:IG-DMR	0.4484537	-0.103656391	0.5521101	0.0445735	0.4183897	0.6858305
11	2019606	cg15886040	H19/IGF2:IG-DMR	0.5181898	-0.077743891	0.5959337	0.0337425	0.494706	0.6971613
11	2019624	cg10154633	H19/IGF2:IG-DMR	0.5347043	-0.056555964	0.5912603	0.0227289	0.5230736	0.6594469
11	2019626	cg26469586	H19/IGF2:IG-DMR	0.49009	-0.041942482	0.5320325	0.0154895	0.4855639	0.578501
11	2019655	cg24409677	H19/IGF2:IG-DMR	0.5525873	-0.057290827	0.6098781	0.0239599	0.5379985	0.6817578
11	2019667	cg16303279	H19/IGF2:IG-DMR	0.5099586	-0.067526618	0.5774852	0.0199298	0.5176957	0.6372747
11	2019730	cg02694715	H19/IGF2:IG-DMR	0.5616329	-0.030212236	0.5918451	0.0124428	0.5545167	0.6291736
11	2019732	cg11735853	H19/IGF2:IG-DMR	0.4932342	-0.0472534	0.5404876	0.0176872	0.4874261	0.5935491
11	2019736	cg01977486	H19/IGF2:IG-DMR	0.6284156	-0.048026082	0.6764417	0.0134109	0.636209	0.7166744
11	2019798	cg25838645	H19/IGF2:IG-DMR	0.5102538	-0.072405491	0.5826593	0.022529	0.5150722	0.6502463
11	2019823	cg13581483	H19/IGF2:IG-DMR	0.499142	-0.0538392	0.5529812	0.0189778	0.4960479	0.6099145
11	2019859	cg04088212	H19/IGF2:IG-DMR	0.5454711	-0.095521609	0.6409927	0.0284368	0.5556824	0.7263031
11	2019862	cg01539474	H19/IGF2:IG-DMR	0.5573161	-0.070384391	0.6277005	0.0223662	0.5606018	0.6947991
11	2019930	cg18362496	H19/IGF2:IG-DMR	0.4634848	-0.047015564	0.5105004	0.0161541	0.462038	0.5589627
11	2019943	cg24605090	H19/IGF2:IG-DMR	0.5187417	-0.045548027	0.5642897	0.0197829	0.5049411	0.6236384
11	2020030	cg06749854	H19/IGF2:IG-DMR	0.5422798	-0.075892618	0.6181724	0.0235455	0.5475358	0.688809
11	2020036	cg24510613	H19/IGF2:IG-DMR	0.4968688	-0.071721291	0.5685901	0.0114266	0.5343104	0.6028698
11	2020101	cg16675558	H19/IGF2:IG-DMR	0.7309617	-0.049565764	0.7805275	0.0232568	0.710757	0.8502979
11	2020104	cg03996735	H19/IGF2:IG-DMR	0.6675228	-0.0692315	0.7367543	0.0321494	0.6403061	0.8332025
11	2020118	cg18104242	H19/IGF2:IG-DMR	0.7042579	-0.062684173	0.7669421	0.0289295	0.6801536	0.8537306
11	2020129	cg27300742	H19/IGF2:IG-DMR	0.5401224	-0.066868355	0.6069908	0.0222905	0.5401191	0.6738624
11	2020279	cg25281616	H19/IGF2:IG-DMR	0.5387889	-0.0459793	0.5847682	0.0162511	0.5360148	0.6335216
11	2020286	cg01895612	H19/IGF2:IG-DMR	0.511108	-0.047221445	0.5583294	0.0163814	0.5091854	0.6074735
11	2020296	cg23476401	H19/IGF2:IG-DMR	0.5407081	-0.061499227	0.6022073	0.019215	0.5445622	0.6598525
11	2020314	cg00237904	H19/IGF2:IG-DMR	0.4889621	-0.075836173	0.5647983	0.0254213	0.4885344	0.6410621
11	2020391	cg06765785	H19/IGF2:IG-DMR	0.4244049	-0.075501591	0.4999065	0.0165034	0.4503962	0.5494168
11	2020417	cg25821896	H19/IGF2:IG-DMR	0.5398909	-0.056730073	0.596621	0.0135794	0.5558827	0.6373593
11	2020496	cg25574978	H19/IGF2:IG-DMR	0.5429146	-0.033115118	0.5760297	0.0191124	0.5186927	0.6333668
11	2020537	cg18454954	H19/IGF2:IG-DMR	0.4630322	-0.030151364	0.4931836	0.0141995	0.450585	0.5357821
11	2020549	cg25579157	H19/IGF2:IG-DMR	0.5131997	-0.025158327	0.538358	0.0122103	0.5017271	0.5749889
11	2020556	cg02886509	H19/IGF2:IG-DMR	0.4248658	-0.078541127	0.5034069	0.0267228	0.4232386	0.5835753
11	2020560	cg02657360	H19/IGF2:IG-DMR	0.4086723	-0.048219227	0.4568915	0.017194	0.4053096	0.5084735
11	2021103	cg27372170	H19/IGF2:IG-DMR	0.626014	-0.000251982	0.626266	0.0203235	0.5652956	0.6872364
11	2021243	cg22259242	H19/IGF2:IG-DMR	0.5562934	0.007717782	0.5485756	0.021236	0.4848675	0.6122837
11	2021658	cg19177307	H19/IGF2:IG-DMR	0.5928013	-0.010199264	0.6030006	0.0135958	0.5622133	0.6437879
11	2021915	cg01585333	H19/IGF2:IG-DMR	0.5637922	-0.0050237	0.5688159	0.0253607	0.4927338	0.644898
11	2022021	cg06982169	H19/IGF2:IG-DMR	0.6895005	0.023920573	0.6655799	0.0861865	0.4070206	0.9241393
11	2022324	cg16574793	H19/IGF2:IG-DMR	0.5858428	-0.008404245	0.594247	0.0201118	0.5339117	0.6545824
11	2022386	cg09452478	H19/IGF2:IG-DMR	0.5383947	0.003840682	0.534554	0.0194976	0.4760614	0.5930467
11	2023450	cg02864690	H19/IGF2:IG-DMR	0.535144	-0.047105018	0.582249	0.0209908	0.5192767	0.6452213
11	2023865	cg20891060	H19/IGF2:IG-DMR	0.6372507	-0.013193345	0.650444	0.0235503	0.5797932	0.7210949
11	2024126	cg19024989	H19/IGF2:IG-DMR	0.6035669	-0.014015936	0.6175828	0.0151307	0.5721908	0.6629748
11	2153991	cg02807948	IGF2:Ex9-DMR	0.4889074	-0.028077827	0.5169852	0.0187071	0.4608638	0.5731067
11	2154074	cg12528452	IGF2:Ex9-DMR	0.5939729	-0.0405188	0.6344917	0.0250718	0.5592763	0.7097071
11	2154113	cg13165070	IGF2:Ex9-DMR	0.3622051	-0.038136545	0.4003416	0.0245118	0.3268063	0.473877
11	2154132	cg11717189	IGF2:Ex9-DMR	0.3755741	-0.062633218	0.4382073	0.0328216	0.3397426	0.5366721
11	2154255	cg07096953	IGF2:Ex9-DMR	0.2581349	-0.073650827	0.3317857	0.018799	0.2753888	0.3881827
11	2154386	cg02613624	IGF2:Ex9-DMR	0.5848997	-0.037908155	0.6228079	0.0133586	0.5827321	0.6628836
11	2154432	cg22956483	IGF2:Ex9-DMR	0.3146766	-0.038959255	0.3536359	0.0227773	0.2853038	0.4219679
11	2154638	cg26401390	IGF2:Ex9-DMR	0.5134489	-0.0323066	0.5457555	0.0108869	0.5130949	0.5784161
11	2154934	cg05384664	IGF2:Ex9-DMR	0.597232	-0.028623609	0.6258556	0.0162678	0.5770523	0.674659
11	2154952	cg00570518	IGF2:Ex9-DMR	0.5579205	-0.026350764	0.5842713	0.0149616	0.5393864	0.6291561
11	2168625	cg19642877	IGF2:alt-TSS	0.539409	-0.035477155	0.5748862	0.0179138	0.5211447	0.6286276

11	2720229	cg27119222	KCNQ1OT1:TSS-DMR	0.6765351	0.023616418	0.6529187	0.0176419	0.5999929	0.7058445
11	2720463	cg00000924	KCNQ1OT1:TSS-DMR	0.5928181	0.023719845	0.5690983	0.012816	0.5306501	0.6075464
11	2720810	cg11297256	KCNQ1OT1:TSS-DMR	0.6320696	-0.001766464	0.6338361	0.0211469	0.5703953	0.6972768
11	2721207	cg14392746	KCNQ1OT1:TSS-DMR	0.5702896	0.019553455	0.5507361	0.0235184	0.4801808	0.6212915
11	2721243	cg12077660	KCNQ1OT1:TSS-DMR	0.5465612	0.011238627	0.5353226	0.0195477	0.4766795	0.5939656
11	2721248	cg03401726	KCNQ1OT1:TSS-DMR	0.550069	-0.005429291	0.5554983	0.0146718	0.5114829	0.5995137
11	2721336	cg16739686	KCNQ1OT1:TSS-DMR	0.5770524	0.020571236	0.5564812	0.0274027	0.4742731	0.6386892
11	2721351	cg08359167	KCNQ1OT1:TSS-DMR	0.5202184	-0.0058531	0.5260715	0.0168578	0.4754981	0.5766449
11	2721366	cg08446215	KCNQ1OT1:TSS-DMR	0.6072333	0.034565373	0.5726679	0.03353	0.4720778	0.6732581
11	2721383	cg26104781	KCNQ1OT1:TSS-DMR	0.6221972	0.035098436	0.5870988	0.0319024	0.4913917	0.6828058
11	2721409	cg02219360	KCNQ1OT1:TSS-DMR	0.5727867	0.0303151	0.5424716	0.0312946	0.4485879	0.6363553
11	2721428	cg20699737	KCNQ1OT1:TSS-DMR	0.5746276	0.025712236	0.5489154	0.0336739	0.4478937	0.649937
11	2721437	cg26547719	KCNQ1OT1:TSS-DMR	0.5361654	0.0138678	0.5222976	0.0260048	0.4442832	0.600312
11	2721480	cg07595203	KCNQ1OT1:TSS-DMR	0.4723673	0.032914209	0.4394531	0.0280447	0.3553189	0.5235873
11	2721591	cg09518720	KCNQ1OT1:TSS-DMR	0.6212664	0.0193476	0.6019188	0.0369198	0.4911593	0.7126783
11	2721610	cg14958441	KCNQ1OT1:TSS-DMR	0.5249326	0.015310773	0.5096218	0.0152793	0.4637839	0.5554597
11	2721619	cg27323091	KCNQ1OT1:TSS-DMR	0.555064	0.014202955	0.540861	0.0142402	0.4981404	0.5835817
11	2721632	cg01873334	KCNQ1OT1:TSS-DMR	0.6767731	0.001541264	0.6752318	0.0200149	0.6151871	0.7352766
11	2721799	cg05816130	KCNQ1OT1:TSS-DMR	0.627732	0.024196509	0.6035355	0.0136398	0.5626161	0.6444549
11	2721817	cg14243741	KCNQ1OT1:TSS-DMR	0.6537364	0.018601982	0.6351344	0.0131006	0.5958325	0.6744363
11	2721857	cg21137515	KCNQ1OT1:TSS-DMR	0.6299779	0.022859918	0.607118	0.011374	0.5729959	0.64124
11	2721866	cg05740879	KCNQ1OT1:TSS-DMR	0.6521242	0.013081918	0.6390423	0.0167219	0.5888767	0.6892079
11	2721952	cg01893176	KCNQ1OT1:TSS-DMR	0.5199384	0.011288764	0.5086496	0.0126094	0.4708215	0.5464778
11	2721961	cg11993252	KCNQ1OT1:TSS-DMR	0.6060042	0.013550227	0.592454	0.0128898	0.5537847	0.6311233
11	2722062	cg11666921	KCNQ1OT1:TSS-DMR	0.472666	-0.019569555	0.4922356	0.0239907	0.4202634	0.5642077
11	2722073	cg26094482	KCNQ1OT1:TSS-DMR	0.4604272	0.000552791	0.4598744	0.0140404	0.4177533	0.5019955
11	2722082	cg03422070	KCNQ1OT1:TSS-DMR	0.6225312	0.013122673	0.6094085	0.0105791	0.5776712	0.6411459
11	2722084	cg15651941	KCNQ1OT1:TSS-DMR	0.5735396	0.014246336	0.5592933	0.0178588	0.5057169	0.6128697
11	2722086	cg25306939	KCNQ1OT1:TSS-DMR	0.5877236	0.015602182	0.5721214	0.0110998	0.5388221	0.6054207
11	2722119	cg06288089	KCNQ1OT1:TSS-DMR	0.5328559	0.0339417	0.4989142	0.016956	0.4480463	0.5497821
11	2722195	cg02798157	KCNQ1OT1:TSS-DMR	0.5845579	0.019212891	0.565345	0.0161721	0.5168286	0.6138614
11	2722258	cg27604721	KCNQ1OT1:TSS-DMR	0.6198991	-0.006661136	0.6265602	0.0134764	0.5861311	0.6669894
13	48892551	cg27182551	RB1:Int2-DMR	0.6723116	-0.019747655	0.6920593	0.0268439	0.6115276	0.7725909
13	48892948	cg19427472	RB1:Int2-DMR	0.6520948	-0.002001255	0.6540961	0.0203149	0.5931514	0.7150407
13	48893174	cg13431205	RB1:Int2-DMR	0.536038	0.015767318	0.5202707	0.0175099	0.4677408	0.5728005
13	48893376	cg03085377	RB1:Int2-DMR	0.5190378	0.027084245	0.4919536	0.0232828	0.4221051	0.561802
13	48893727	cg18481241	RB1:Int2-DMR	0.5354028	0.040281255	0.4951215	0.0168259	0.4446439	0.5455992
13	48893968	cg16153267	RB1:Int2-DMR	0.4030024	0.027285873	0.3757165	0.0122951	0.3388312	0.4126019
13	48894214	cg25676335	RB1:Int2-DMR	0.663223	-0.002296991	0.66552	0.0082383	0.6408051	0.6902349
13	48894382	cg00059930	RB1:Int2-DMR	0.551289	0.007971491	0.5433175	0.0133144	0.5033743	0.5832607
13	48894595	cg26119796	RB1:Int2-DMR	0.5027417	0.011487618	0.4912541	0.0161168	0.4429038	0.5396044
13	48895173	cg19254235	RB1:Int2-DMR	0.5832275	0.005971773	0.5772557	0.0205775	0.5155233	0.6389882
13	48895241	cg26740195	RB1:Int2-DMR	0.5345027	0.008131145	0.5263716	0.0104616	0.4949869	0.5577562
13	48895318	cg27026713	RB1:Int2-DMR	0.6097808	0.000683764	0.609097	0.0228175	0.5406444	0.6775496
13	48895478	cg07880715	RB1:Int2-DMR	0.659754	0.002178491	0.6575755	0.0164907	0.6081035	0.7070475
14	101290556	cg00764369	MEG3:TSS-DMR	0.4933982	-0.221038555	0.7144368	0.0360241	0.6063646	0.8225089
14	101290717	cg10515315	MEG3:TSS-DMR	0.3203133	-0.233634745	0.553948	0.0149889	0.5089812	0.5989149
14	101290867	cg15991553	MEG3:TSS-DMR	0.3381327	-0.256437945	0.5945706	0.0190425	0.5374431	0.6516982
14	101291068	cg23870378	MEG3:TSS-DMR	0.387756	-0.188555791	0.5763118	0.0173325	0.5243143	0.6283093
14	101291100	cg14034270	MEG3:TSS-DMR	0.6699093	-0.0975098	0.7674191	0.0209811	0.7044759	0.8303623
14	101291136	cg16567044	MEG3:TSS-DMR	0.5176239	-0.041804609	0.5594285	0.0202217	0.4987634	0.6200937
14	101291180	cg10540302	MEG3:TSS-DMR	0.4652876	-0.0697831	0.5350707	0.0167058	0.4849533	0.5851881
14	101291288	cg02313552	MEG3:TSS-DMR	0.5548627	-0.1431661	0.6980288	0.0325078	0.6005054	0.7955522
14	101291410	cg02982753	MEG3:TSS-DMR	0.3459208	-0.214567636	0.5604884	0.0110223	0.5274215	0.5935553
14	101291440	cg02888166	MEG3:TSS-DMR	0.4839814	-0.153481127	0.6374625	0.0223067	0.5705424	0.7043826
14	101291459	cg24089209	MEG3:TSS-DMR	0.4285262	-0.168896218	0.5974224	0.0146129	0.5535838	0.641261
14	101291469	cg16738180	MEG3:TSS-DMR	0.5242405	-0.0998897	0.6241302	0.0200425	0.5640027	0.6842577
14	101291500	cg19643803	MEG3:TSS-DMR	0.3961833	-0.187910245	0.5840935	0.0132815	0.5442492	0.6239379
14	101291571	cg10943497	MEG3:TSS-DMR	0.4886064	-0.219085882	0.7076923	0.0256341	0.6307899	0.7845947
14	101291602	cg10065153	MEG3:TSS-DMR	0.4195171	-0.187903018	0.6074201	0.0318687	0.5118142	0.7030261
14	101291687	cg09280976	MEG3:TSS-DMR	0.5187946	-0.1173891	0.6361837	0.0153665	0.5900842	0.6822832
14	101291829	cg22940816	MEG3:TSS-DMR	0.3245012	-0.206601636	0.5311028	0.0124446	0.4937689	0.5684368
14	101291856	cg14070323	MEG3:TSS-DMR	0.3015412	-0.306436482	0.6079777	0.0276158	0.5251302	0.6908252
14	101291891	cg14119337	MEG3:TSS-DMR	0.2470936	-0.228216355	0.47531	0.0203685	0.4142044	0.5364155
14	101291933	cg20952167	MEG3:TSS-DMR	0.2532074	-0.253528709	0.5067361	0.0292058	0.4191186	0.5943537
14	101291997	cg12967319	MEG3:TSS-DMR	0.2879412	-0.245852082	0.5337933	0.0130733	0.4945734	0.5730131
14	101292128	cg04304932	MEG3:TSS-DMR	0.294639	-0.290441764	0.5850808	0.0153932	0.538901	0.6312605
14	101292149	cg04576764	MEG3:TSS-DMR	0.2893611	-0.303197373	0.5925585	0.0219305	0.526767	0.6583499
14	101292306	cg25836301	MEG3:TSS-DMR	0.3168771	-0.263782609	0.5806597	0.0130651	0.5414643	0.6198552
14	101292392	cg09926418	MEG3:TSS-DMR	0.3000828	-0.2902658	0.5903486	0.0188025	0.5339412	0.646756
14	101292643	cg23176399	MEG3:TSS-DMR	0.3027038	-0.2261776	0.5288814	0.0143463	0.4858426	0.5719202
14	101292680	cg05711886	MEG3:TSS-DMR	0.3529263	-0.225621464	0.5785478	0.0186152	0.5227021	0.6343934
14	101292872	cg15419911	MEG3:TSS-DMR	0.3084355	-0.286194173	0.5946297	0.0274398	0.5123103	0.676949
14	101293090	cg15373285	MEG3:TSS-DMR	0.3073328	-0.238087718	0.5454205	0.0190259	0.4883429	0.6024981
14	101293450	cg14123427	MEG3:TSS-DMR	0.3390991	-0.189666982	0.5287661	0.0147156	0.4846193	0.5729128
14	101293726	cg15101633	MEG3:TSS-DMR	0.3503993	-0.212651873	0.5630512	0.0157973	0.5156592	0.6104431
14	101293856	cg26374305	MEG3:TSS-DMR	0.3222083	-0.267350127	0.5895584	0.0446374	0.4556461	0.7234707
14	101370989	cg06804177	MEG8:Int2-DMR	0.7367616	0.194870591	0.541891	0.0248057	0.4674738	0.6163082
15	23807180	cg17819168	MKRN3:TSS-DMR	0.7130679	-0.0128483	0.7259162	0.0223405	0.6588947	0.7929377

15	23807234	cg13423098	MKRN3:TSS-DMR	0.5387024	-0.002497545	0.5411999	0.0302095	0.4505714	0.6318285
15	23810163	cg09437135	MKRN3:TSS-DMR	0.8279614	-0.016585827	0.8445472	0.0186841	0.7884948	0.9005996
15	23810238	cg20769842	MKRN3:TSS-DMR	0.7967378	-0.017044882	0.8137827	0.0228523	0.7452257	0.8823397
15	23810280	cg16131766	MKRN3:TSS-DMR	0.8727709	-0.0094258	0.8821967	0.0160446	0.8340628	0.9303306
15	23810334	cg20792895	MKRN3:TSS-DMR	0.904354	-0.018308509	0.9226625	0.0225452	0.8550268	0.9902982
15	23810378	cg05952543	MKRN3:TSS-DMR	0.887573	-0.003001	0.890574	0.0146123	0.8467372	0.9344108
15	23810652	cg27046052	MKRN3:TSS-DMR	0.790598	-0.001021445	0.7916194	0.1095853	0.4628635	1.1203754
15	23810861	cg11100640	MKRN3:TSS-DMR	0.8267598	-0.024028182	0.850788	0.020767	0.7884869	0.9130891
15	23811205	cg23234999	MKRN3:TSS-DMR	0.8116753	-0.026243491	0.8379188	0.0194173	0.7796668	0.8961708
15	23811369	cg19903927	MKRN3:TSS-DMR	0.7387767	-0.047672436	0.7864491	0.0332247	0.686775	0.8861232
15	23812334	cg07760796	MKRN3:TSS-DMR	0.8656992	-0.013630527	0.8793297	0.0112619	0.8455441	0.9131153
15	23892574	cg20747630	MAGEL2:TSS-DMR	0.6145458	-0.006153636	0.6206994	0.0211188	0.557343	0.6840558
15	23892656	cg04142555	MAGEL2:TSS-DMR	0.630088	0.021740645	0.6083474	0.0239748	0.5364229	0.6802718
15	23892770	cg14432018	MAGEL2:TSS-DMR	0.6525091	0.029203691	0.6233054	0.0124788	0.585869	0.6607418
15	23893025	cg19961998	MAGEL2:TSS-DMR	0.5164475	0.040307845	0.4761397	0.0289358	0.3893323	0.562947
15	23893040	cg18253764	MAGEL2:TSS-DMR	0.5062156	0.006951373	0.4992642	0.0159661	0.4513658	0.5471627
15	23893742	cg22872376	MAGEL2:TSS-DMR	0.4509062	0.004659282	0.4462469	0.030316	0.3552989	0.537195
15	23931451	cg06743811	NDN:TSS-DMR	0.6835766	-0.023105391	0.706682	0.0108894	0.6740139	0.73935
15	23931674	cg18602919	NDN:TSS-DMR	0.6418664	-0.029975955	0.6718424	0.0300299	0.5817526	0.7619321
15	23932370	cg12532169	NDN:TSS-DMR	0.5037931	0.043529045	0.4602641	0.0191151	0.4029189	0.5176092
15	23932373	cg13828758	NDN:TSS-DMR	0.5729367	0.031742618	0.5411941	0.0161164	0.4928449	0.5895433
15	23932397	cg22694067	NDN:TSS-DMR	0.5078565	0.052319527	0.455537	0.0286745	0.3695134	0.5415606
15	23932412	cg26725891	NDN:TSS-DMR	0.5807614	0.047154218	0.5336072	0.0296383	0.4446923	0.622522
15	23932620	cg18552939	NDN:TSS-DMR	0.4439768	0.060751227	0.3832256	0.0237476	0.3119829	0.4544682
15	23932758	cg01989224	NDN:TSS-DMR	0.6187767	-0.026192918	0.6449696	0.031092	0.5516936	0.7382457
15	24346942	cg09985260		0.5378052	0.009129864	0.5286753	0.0262845	0.4498217	0.607529
15	24347063	cg14555733		0.4887022	-0.0056165	0.4943187	0.0269118	0.4135833	0.5750541
15	24672032	cg26288595		0.6514387	0.050848282	0.6005904	0.0585726	0.4248726	0.7763082
15	24672285	cg08449389		0.2118048	-0.0231973	0.2350021	0.0492848	0.0871478	0.3828564
15	24672463	cg22261467		0.3756776	-0.018296327	0.3939739	0.0253999	0.3177742	0.4701736
15	24672531	cg26708976		0.4641632	0.001728809	0.4624344	0.0230064	0.3934153	0.5314535
15	24722944	cg00374016		0.4854053	0.024668473	0.4607368	0.0559765	0.2928074	0.6286662
15	25018132	cg15298722		0.3041247	0.053257164	0.2508675	0.0283566	0.1657979	0.3359372
15	25018227	cg13495693		0.4861854	0.0272364	0.458949	0.0302184	0.3682938	0.5496042
15	25018302	cg00229754		0.3577048	0.005085491	0.3526193	0.0287714	0.266305	0.4389336
15	25018449	cg20707630		0.2947138	0.014007318	0.2807065	0.0221555	0.2142399	0.3471731
15	25068564	cg22491305	SNRPN:alt-TSS-DMR	0.837501	0.027615464	0.8098855	0.0225052	0.7423699	0.8774012
15	25068738	cg11265941	SNRPN:alt-TSS-DMR	0.5398623	-0.016871709	0.556734	0.0172363	0.505025	0.608443
15	25068754	cg11826663	SNRPN:alt-TSS-DMR	0.5959485	0.005854018	0.5900945	0.0167548	0.5398301	0.6403588
15	25068757	cg24785225	SNRPN:alt-TSS-DMR	0.6062745	0.007736391	0.5985381	0.0128984	0.5598429	0.6372333
15	25068763	cg10271763	SNRPN:alt-TSS-DMR	0.5870005	0.002329973	0.5846705	0.0156123	0.5378335	0.6315076
15	25068769	cg27304225	SNRPN:alt-TSS-DMR	0.611575	0.013347945	0.5982271	0.0151036	0.5529163	0.6435378
15	25068790	cg19803984	SNRPN:alt-TSS-DMR	0.5238163	0.002247855	0.5215684	0.0192635	0.4637778	0.5793591
15	25068850	cg16321029	SNRPN:alt-TSS-DMR	0.5109238	-8.71909E-05	0.511011	0.0207495	0.4487624	0.5732596
15	25069376	cg26033681	SNRPN:alt-TSS-DMR	0.6278529	0.030479327	0.5973736	0.0284468	0.5120331	0.682714
15	25093244	cg25978208	SNRPN:Int1-DMR1	0.4967384	-0.037085809	0.5338242	0.0294034	0.4456141	0.6220343
15	25093366	cg02171545	SNRPN:Int1-DMR1	0.3679143	-0.018894245	0.3868085	0.0584162	0.2115599	0.5620572
15	25093456	cg02305723	SNRPN:Int1-DMR1	0.4670864	-0.007443836	0.4745302	0.0300437	0.3843991	0.5646614
15	25093521	cg22678136	SNRPN:Int1-DMR1	0.44306	-0.030897864	0.4739579	0.0468237	0.3334866	0.6144291
15	25094139	cg13887424	SNRPN:Int1-DMR1	0.8803242	0.002657036	0.8776672	0.0094947	0.8491831	0.9061512
15	25123287	cg27644292	SNRPN:Int1-DMR2	0.611891	0.011494818	0.6003962	0.0239534	0.5285361	0.6722563
15	25123381	cg08560373	SNRPN:Int1-DMR2	0.4148766	0.045029964	0.3698466	0.0365542	0.2601842	0.4795091
15	25123491	cg25657700	SNRPN:Int1-DMR2	0.6176287	-0.003442218	0.6210709	0.0562785	0.4522353	0.7899065
15	25123549	cg24993443	SNRPN:Int1-DMR2	0.518802	0.038429818	0.4803722	0.024435	0.4070672	0.5536772
15	25123688	cg01786704	SNRPN:Int1-DMR2	0.4492505	-0.000307545	0.449558	0.061647	0.2646172	0.6344989
15	25123731	cg21870668	SNRPN:Int1-DMR2	0.5387175	0.014954182	0.5237633	0.0250447	0.4486293	0.5988973
15	25124213	cg21061553		0.8566232	0.000905491	0.8557177	0.0127608	0.8174354	0.8940001
15	25199270	cg02152271		0.8516996	-0.0090359	0.8607355	0.0077794	0.8373974	0.8840736
15	25199713	cg12298755		0.7953832	0.001684291	0.7936989	0.0208111	0.7312655	0.8561323
15	25200253	cg18506672	SNRUF:TSS-DMR	0.5532163	0.008042082	0.5451742	0.0251663	0.4696752	0.6206732
15	25200406	cg02125271	SNRUF:TSS-DMR	0.70816	0.0247238	0.6834362	0.0216621	0.6184499	0.7484225
15	25200490	cg26875073	SNRUF:TSS-DMR	0.5389802	-0.010877882	0.5498581	0.0399261	0.4300797	0.6696365
15	25201020	cg22159025	SNRUF:TSS-DMR	0.5798151	0.073338636	0.5064765	0.0309746	0.4135526	0.5994004
15	25201224	cg22555495	SNRUF:TSS-DMR	0.54295	0.001864736	0.5410853	0.0182667	0.4862853	0.5958853
15	25201429	cg01614564	SNRUF:TSS-DMR	0.6026339	-0.000221282	0.6028552	0.0151097	0.557526	0.6481843
15	25201732	cg13073261	SNRUF:TSS-DMR	0.584655	0.040788545	0.5438665	0.0291909	0.4562938	0.6314391
15	99408636	cg19322380		0.6852337	0.020777282	0.6644564	0.0314578	0.5700831	0.7588297
15	99408804	cg21746425		0.5568296	0.029042327	0.5277873	0.033785	0.4264323	0.6291423
15	99408958	cg12553689	IGF1R:Int2-DMR	0.6223594	0.029163718	0.5931957	0.021855	0.5276307	0.6587606
15	99409194	cg07615383	IGF1R:Int2-DMR	0.5036473	0.016991973	0.4866553	0.02905	0.3995053	0.5738054
15	99409360	cg00098799	IGF1R:Int2-DMR	0.5784082	0.030919255	0.5474889	0.0425904	0.4197177	0.6752602
15	99409411	cg13812291	IGF1R:Int2-DMR	0.6007113	-0.007219045	0.6079303	0.0316384	0.5130151	0.7028456
15	99409506	cg03380198	IGF1R:Int2-DMR	0.5966363	0.025575464	0.5710608	0.0332512	0.4713073	0.6708144
15	99409942	cg25404375		0.603549	0.051461	0.552088	0.0407926	0.4297101	0.6744659
15	99409948	cg17074431		0.6934785	0.027178445	0.6663001	0.0226489	0.5983534	0.7342467
15	99409957	cg11544420		0.6373245	0.026826436	0.6104981	0.025338	0.5344839	0.6865122
16	3481970	cg02880119	ZNF597:3'-DMR	0.6557009	0.002174145	0.6535268	0.0318113	0.5580928	0.7489607
16	3482078	cg04027059	ZNF597:3'-DMR	0.6799327	0.0066465	0.6732862	0.0313165	0.5793366	0.7672358
16	3493133	cg06410284	ZNF597:TSS-DMR	0.508882	0.006225409	0.5026566	0.021923	0.4368877	0.5684255

16	3493336	cg09596866	ZNF597:TSS-DMR	0.4287093	0.014967627	0.4137417	0.0485419	0.268116	0.5593673
16	3493423	cg10482201	ZNF597:TSS-DMR	0.5577776	0.017631118	0.5401465	0.0452009	0.4045437	0.6757493
16	3493428	cg24333473	ZNF597:TSS-DMR	0.3506653	0.008490991	0.3421743	0.0419329	0.2163757	0.4679729
16	3493440	cg03896694	ZNF597:TSS-DMR	0.5616907	0.042919327	0.5187714	0.0456818	0.381726	0.6558167
16	3493534	cg27136643	ZNF597:TSS-DMR	0.5853598	0.009882518	0.5754773	0.0308661	0.4828788	0.6680757
16	3493614	cg03933495	ZNF597:TSS-DMR	0.4540284	0.018342027	0.4356864	0.0338315	0.3341919	0.5371809
16	3493681	cg27460547	ZNF597:TSS-DMR	0.5296268	0.023295518	0.5063313	0.032035	0.4102264	0.6024362
16	3493815	cg26918062	ZNF597:TSS-DMR	0.5023755	0.039838382	0.4625371	0.025015	0.387492	0.5375823
16	3493997	cg14654875	ZNF597:TSS-DMR	0.5486475	0.014621027	0.5340265	0.0215646	0.4693328	0.5987202
16	3494094	cg10406676	ZNF597:TSS-DMR	0.5552104	0.028903055	0.5263073	0.0300656	0.4361106	0.6165041
16	3494155	cg06121207	ZNF597:TSS-DMR	0.5566941	0.011789064	0.544905	0.0278651	0.4613098	0.6285003
19	54040774	cg20646939	ZNF331:alt-TSS-DMR1	0.5251881	0.008432727	0.5167554	0.0155426	0.4701276	0.5633832
19	54040813	cg06855497	ZNF331:alt-TSS-DMR1	0.5647159	0.023487882	0.541228	0.0147252	0.4970525	0.5854035
19	54040818	cg15576576	ZNF331:alt-TSS-DMR1	0.6025573	0.016118509	0.5864388	0.0179447	0.5326046	0.640273
19	54041163	cg22475353	ZNF331:alt-TSS-DMR1	0.3821808	0.000746709	0.3814341	0.0239488	0.3095876	0.4532806
19	54041251	cg27296330	ZNF331:alt-TSS-DMR1	0.4558443	0.026087618	0.4297567	0.0179929	0.3757779	0.4837355
19	54041303	cg10023249	ZNF331:alt-TSS-DMR1	0.2907017	0.030033009	0.2606687	0.0242701	0.1878583	0.3334791
19	54041308	cg15607708	ZNF331:alt-TSS-DMR1	0.3179553	-0.001033236	0.3189885	0.0231213	0.2496245	0.3883525
19	54041329	cg04522821	ZNF331:alt-TSS-DMR1	0.5053216	0.026632818	0.4786888	0.0205465	0.4170493	0.5403282
19	54041398	cg05338009	ZNF331:alt-TSS-DMR1	0.5042208	0.034494027	0.4697268	0.0565183	0.300172	0.6392815
19	54041856	cg03499639	ZNF331:alt-TSS-DMR1	0.563082	0.040008118	0.5230739	0.0180148	0.4690295	0.5771182
19	54041999	cg25409185	ZNF331:alt-TSS-DMR1	0.5353812	0.018523118	0.5168581	0.0344585	0.4134826	0.6202335
19	54042165	cg15545827	ZNF331:alt-TSS-DMR1	0.5841751	0.018508718	0.5656664	0.022078	0.4994325	0.6319003
19	54057208	cg06860848	ZNF331:alt-TSS-DMR2	0.5415506	0.013064209	0.5284864	0.0159111	0.480753	0.5762198
19	54057415	cg03113572	ZNF331:alt-TSS-DMR2	0.6217358	0.025963409	0.5957724	0.0172955	0.5438859	0.6476588
19	54057705	cg19696891	ZNF331:alt-TSS-DMR2	0.4597017	0.017346964	0.4423547	0.0229544	0.3734917	0.5112178
19	54058085	cg20071427	ZNF331:alt-TSS-DMR2	0.6032714	0.007832427	0.595439	0.0201831	0.5348898	0.6559882
19	57349204	cg15473155	PEG3:TSS-DMR	0.6082093	-0.004762764	0.6129721	0.020736	0.5507642	0.67518
19	57349679	cg19041006	PEG3:TSS-DMR	0.6030949	0.011673109	0.5914218	0.0211014	0.5281177	0.6547259
19	57349709	cg17663463	PEG3:TSS-DMR	0.6515793	0.005722536	0.6458568	0.0147654	0.6015606	0.6901529
19	57349815	cg19771589	PEG3:TSS-DMR	0.5810561	0.0056748	0.5753813	0.0133477	0.5353382	0.6154244
19	57350004	cg02793099	PEG3:TSS-DMR	0.5593412	0.002823445	0.5565178	0.0163649	0.507423	0.6056125
19	57350096	cg01656470	PEG3:TSS-DMR	0.6571538	0.024323064	0.6328307	0.0182256	0.5781538	0.6875076
19	57350292	cg27519373	PEG3:TSS-DMR	0.7037165	0.029196282	0.6745202	0.0174091	0.622293	0.7267475
19	57350313	cg18516833	PEG3:TSS-DMR	0.7435693	-0.003220173	0.7467895	0.0161973	0.6981976	0.7953814
19	57350503	cg07310951	PEG3:TSS-DMR	0.5527029	0.006103109	0.5465998	0.0199257	0.4868227	0.6063768
19	57350846	cg10719746	PEG3:TSS-DMR	0.671581	0.007652991	0.663928	0.0252992	0.5880303	0.7398257
19	57351213	cg20897667	PEG3:TSS-DMR	0.5875886	-0.002915355	0.590504	0.0164873	0.5410422	0.6399657
19	57351322	cg02478023	PEG3:TSS-DMR	0.6108391	0.050291773	0.5605473	0.0246034	0.4867372	0.6343574
19	57351440	cg22354595	PEG3:TSS-DMR	0.5955085	0.011420645	0.5840879	0.0223625	0.5170003	0.6511754
19	57351642	cg19335327	PEG3:TSS-DMR	0.5867526	0.019531609	0.567221	0.0168082	0.5167963	0.6176457
19	57351791	cg14849423	PEG3:TSS-DMR	0.6352021	0.018096691	0.6171054	0.0133809	0.5769627	0.6572481
19	57352014	cg15777825	PEG3:TSS-DMR	0.5343242	0.016527955	0.5177962	0.019635	0.4588913	0.5767012
19	57352018	cg10204755	PEG3:TSS-DMR	0.595185	0.023439227	0.5717458	0.0184954	0.5162595	0.627232
19	57352021	cg12205903	PEG3:TSS-DMR	0.5212312	0.027179591	0.4940516	0.020458	0.4326776	0.5554256
19	57352074	cg26349266	PEG3:TSS-DMR	0.6014848	0.029829155	0.5716556	0.0161739	0.5231338	0.6201774
19	57352134	cg02162069	PEG3:TSS-DMR	0.5550399	0.030037845	0.5250021	0.011936	0.489194	0.5608101
19	57352142	cg13960339	PEG3:TSS-DMR	0.6208561	0.012101664	0.6087544	0.0157104	0.5616234	0.6558855
19	57352176	cg13369939	PEG3:TSS-DMR	0.6370822	0.015595436	0.6214868	0.0189211	0.5647234	0.6782501
19	57352185	cg20628335	PEG3:TSS-DMR	0.5574216	0.008490245	0.5489314	0.013336	0.5089234	0.5889393
19	57352252	cg22294267	PEG3:TSS-DMR	0.6463314	0.019949291	0.6263821	0.0138861	0.5847238	0.6680404
19	57352269	cg19098268	PEG3:TSS-DMR	0.6026353	0.033374545	0.5692608	0.0124172	0.5320092	0.6065123
19	57352465	cg24844423	PEG3:TSS-DMR	0.5988944	-0.006736891	0.6056313	0.0218519	0.5400757	0.6711869
19	57352519	cg15678121	PEG3:TSS-DMR	0.6381742	0.0112412	0.626933	0.0195622	0.5682464	0.6856196
19	57352542	cg12032885	PEG3:TSS-DMR	0.4373327	0.001411009	0.4359217	0.0224683	0.3685167	0.5033267
19	57352584	cg25458871	PEG3:TSS-DMR	0.5961241	0.033706555	0.5624175	0.0144932	0.5189379	0.6058972
19	57352657	cg18668753	PEG3:TSS-DMR	0.6429652	-3.94545E-06	0.6429691	0.0153512	0.5969156	0.6890227
19	57352686	cg18706888	PEG3:TSS-DMR	0.5971841	-0.000316945	0.597501	0.0213221	0.5335348	0.6614673
19	57352723	cg06652523	PEG3:TSS-DMR	0.5899636	0.002635845	0.5873278	0.0131695	0.5478193	0.6268363
19	57352729	cg01054891	PEG3:TSS-DMR	0.4912651	-0.024915764	0.5161809	0.0129786	0.4772451	0.5551167
19	57352746	cg18644235	PEG3:TSS-DMR	0.7269234	-0.010160436	0.7370838	0.0161348	0.6886794	0.7854883
19	57352785	cg13374648	PEG3:TSS-DMR	0.5505705	0.001770582	0.5487999	0.0106557	0.5168327	0.5807672
19	57352807	cg26917367	PEG3:TSS-DMR	0.7008471	-0.002292009	0.7031391	0.0201482	0.6426946	0.7635836
19	57353128	cg22927979	PEG3:TSS-DMR	0.7785191	-0.004283955	0.7828031	0.0094499	0.7544534	0.8111527
20	30134929	cg25359645	MCTS2P:TSS-DMR	0.5053984	0.020692936	0.4847055	0.0194508	0.4263531	0.5430579
20	30134973	cg24607140	MCTS2P:TSS-DMR	0.6328614	0.026549082	0.6063123	0.0113989	0.5721155	0.6405091
20	30135108	cg06000530	MCTS2P:TSS-DMR	0.5559115	0.027477509	0.528434	0.0234498	0.4580845	0.5987835
20	30135124	cg19617948	MCTS2P:TSS-DMR	0.5653849	0.028227718	0.5371572	0.0285987	0.451361	0.6229534
20	30135144	cg15815607	MCTS2P:TSS-DMR	0.5442942	0.029429482	0.5148647	0.0233513	0.4448107	0.5849187
20	30135149	cg25645178	MCTS2P:TSS-DMR	0.4996886	0.0304221	0.4692665	0.0270991	0.3879691	0.5505639
20	30135158	cg17840843	MCTS2P:TSS-DMR	0.4362545	0.0296568	0.4065977	0.0284374	0.3212856	0.4919098
20	30135177	cg02146091	MCTS2P:TSS-DMR	0.535454	0.026969073	0.5084849	0.0275603	0.4258041	0.5911658
20	30135291	cg14175568	MCTS2P:TSS-DMR	0.5066389	0.015255055	0.4913838	0.0189318	0.4345883	0.5481794
20	30135362	cg20129782	MCTS2P:TSS-DMR	0.4731884	0.003233336	0.4699551	0.0168398	0.4194356	0.5204745
20	36148604	cg23757721	NNAT:TSS-DMR	0.6905107	-0.020107727	0.7106184	0.0190106	0.6535866	0.7676503
20	36148615	cg26083330	NNAT:TSS-DMR	0.6509374	-0.029627455	0.6805649	0.0125793	0.6428269	0.7183028
20	36148620	cg22551578	NNAT:TSS-DMR	0.5157267	-0.035595809	0.5513225	0.0311995	0.4577239	0.6449211
20	36148642	cg04489586	NNAT:TSS-DMR	0.6385523	-0.017470464	0.6560228	0.0161792	0.6074853	0.7045602
20	36148672	cg23605670	NNAT:TSS-DMR	0.5737346	-0.006814064	0.5805487	0.0179221	0.5267824	0.6343149

20	36148679	cg12862537	NNAT:TSS-DMR	0.6267028	-0.0094994	0.6362022	0.0254803	0.5597612	0.7126432
20	36148699	cg10981598	NNAT:TSS-DMR	0.5548803	-0.017254627	0.5721349	0.0229191	0.5033777	0.6408922
20	36148738	cg13790727	NNAT:TSS-DMR	0.6748252	-0.023936345	0.6987615	0.0212395	0.6350432	0.7624799
20	36148767	cg20783699	NNAT:TSS-DMR	0.6492836	-0.021239073	0.6705227	0.024611	0.5966896	0.7443558
20	36148775	cg25712981	NNAT:TSS-DMR	0.7199719	0.007880027	0.7120919	0.0297321	0.6228955	0.8012883
20	36148779	cg03615235	NNAT:TSS-DMR	0.6935411	-0.003498809	0.6970399	0.0279161	0.6132916	0.7807882
20	36148791	cg17643025	NNAT:TSS-DMR	0.694573	0.001057145	0.6935159	0.0277808	0.6101733	0.7768584
20	36148798	cg24762053	NNAT:TSS-DMR	0.8059922	0.010071964	0.7959202	0.0220833	0.7296704	0.8621701
20	36148803	cg14469070	NNAT:TSS-DMR	0.7278794	0.008746773	0.7191326	0.0235491	0.6484854	0.7897798
20	36148860	cg22298088	NNAT:TSS-DMR	0.7368586	0.007959891	0.7288987	0.0223356	0.661892	0.7959055
20	36148928	cg22943498	NNAT:TSS-DMR	0.6499147	-0.006337573	0.6562523	0.0343651	0.553157	0.7593475
20	36148954	cg14765818	NNAT:TSS-DMR	0.6121187	-0.014743545	0.6268622	0.0175986	0.5740664	0.6796581
20	36148961	cg08402058	NNAT:TSS-DMR	0.5706193	-0.019993755	0.5906131	0.0213782	0.5264785	0.6547477
20	36148994	cg15473473	NNAT:TSS-DMR	0.6529029	-0.004330973	0.6572339	0.0245514	0.5835797	0.7308881
20	36149013	cg07156273	NNAT:TSS-DMR	0.6826503	-0.0117502	0.6944005	0.0289513	0.6075465	0.7812545
20	36149022	cg01466133	NNAT:TSS-DMR	0.6801257	-0.002524118	0.6826498	0.0240715	0.6104355	0.7548642
20	36149053	cg24338351	NNAT:TSS-DMR	0.6480232	-0.007545255	0.6555685	0.0173048	0.6036541	0.7074828
20	36149081	cg24675557	NNAT:TSS-DMR	0.6019981	-0.014992436	0.6169905	0.0265152	0.5374449	0.6965362
20	36149112	cg20479660	NNAT:TSS-DMR	0.6456276	-0.0024797	0.6481073	0.0241057	0.5757901	0.7204245
20	36149119	cg22421148	NNAT:TSS-DMR	0.654411	-0.018463645	0.6728746	0.0256026	0.5960668	0.7496825
20	36149121	cg22510412	NNAT:TSS-DMR	0.6721243	-0.012031755	0.6841561	0.0247663	0.609857	0.7584551
20	36149185	cg03061677	NNAT:TSS-DMR	0.6251003	-0.007594318	0.6326946	0.0238318	0.5611991	0.7041902
20	36149188	cg00576435	NNAT:TSS-DMR	0.6403654	0.001154936	0.6392105	0.0191929	0.5816318	0.6967891
20	36149194	cg18433380	NNAT:TSS-DMR	0.6465437	0.000697391	0.6458463	0.0182273	0.5911645	0.7005281
20	36149231	cg16648571	NNAT:TSS-DMR	0.5094878	-0.005458327	0.5149461	0.0241962	0.4423575	0.5875348
20	36149271	cg04810287	NNAT:TSS-DMR	0.5693906	0.027480509	0.5419101	0.0293572	0.4538384	0.6299818
20	36149452	cg25962605	NNAT:TSS-DMR	0.5997887	0.0123788	0.5874099	0.0148515	0.5428553	0.6319645
20	36149455	cg21588305	NNAT:TSS-DMR	0.7026039	-0.0056553	0.7082592	0.0182398	0.6535397	0.7629787
20	36149656	cg13708635	NNAT:TSS-DMR	0.5295927	-0.014686036	0.5442787	0.0143043	0.5013658	0.5871917
20	36149706	cg11174847	NNAT:TSS-DMR	0.5938357	0.000671355	0.5931643	0.0150913	0.5478906	0.6384381
20	36149750	cg23566503	NNAT:TSS-DMR	0.4669992	-0.004222882	0.4712221	0.0198749	0.4115975	0.5308467
20	36150061	cg10642330	NNAT:TSS-DMR	0.5233772	0.062617909	0.4607593	0.0234971	0.3902679	0.5312507
20	42142417	cg22457903	L3MBTL:alt-TSS-DMR	0.5502102	0.017065145	0.5331451	0.0172062	0.4815264	0.5847637
20	42142451	cg20091959	L3MBTL:alt-TSS-DMR	0.583183	0.001538882	0.5816441	0.0143361	0.5386359	0.6246523
20	42142484	cg17091610	L3MBTL:alt-TSS-DMR	0.5740963	0.0026746	0.5714217	0.0130694	0.5322136	0.6106298
20	42142494	cg23626798	L3MBTL:alt-TSS-DMR	0.560655	0.017463982	0.543191	0.0386653	0.4271951	0.659187
20	42142559	cg15388309	L3MBTL:alt-TSS-DMR	0.6145344	0.022799464	0.5917349	0.0176787	0.538699	0.6447709
20	42142596	cg16862791	L3MBTL:alt-TSS-DMR	0.5506218	0.015635864	0.5349859	0.0144571	0.4916145	0.5783574
20	42142671	cg06446163	L3MBTL:alt-TSS-DMR	0.5896057	0.016816882	0.5727888	0.0133767	0.5326588	0.6129188
20	42142673	cg04984575	L3MBTL:alt-TSS-DMR	0.5900688	0.025138627	0.5649302	0.0191666	0.5074305	0.6224298
20	42142699	cg02177317	L3MBTL:alt-TSS-DMR	0.6128479	-0.002758927	0.6156068	0.0171822	0.5640602	0.6671535
20	42142751	cg20529070	L3MBTL:alt-TSS-DMR	0.6098747	0.010181836	0.5996929	0.0162771	0.5508614	0.6485243
20	42142766	cg10360552	L3MBTL:alt-TSS-DMR	0.5740174	0.017186627	0.5568308	0.019134	0.4994288	0.6142327
20	42142784	cg18911945	L3MBTL:alt-TSS-DMR	0.5180553	-0.019321127	0.5373764	0.0244259	0.4640986	0.6106542
20	42142847	cg01877937	L3MBTL:alt-TSS-DMR	0.5706586	0.038080582	0.532578	0.0217112	0.4674445	0.5977115
20	42142852	cg08633313	L3MBTL:alt-TSS-DMR	0.5835131	0.044131409	0.5393817	0.0223832	0.472232	0.6065313
20	42142897	cg12699433	L3MBTL:alt-TSS-DMR	0.5680664	0.037084636	0.5309818	0.0259539	0.4531201	0.6088434
20	42142947	cg11319028	L3MBTL:alt-TSS-DMR	0.5509655	0.015248691	0.5357168	0.0339455	0.4338803	0.6375533
20	42142995	cg22601123	L3MBTL:alt-TSS-DMR	0.601235	0.038025655	0.5632093	0.0237115	0.4920749	0.6343438
20	42143015	cg22330467	L3MBTL:alt-TSS-DMR	0.6316169	0.030540391	0.6010765	0.0338721	0.4994601	0.7026929
20	42143045	cg14306330	L3MBTL:alt-TSS-DMR	0.5408382	0.068223855	0.4726143	0.0319324	0.3768173	0.5684114
20	42143080	cg01071811	L3MBTL:alt-TSS-DMR	0.6518515	0.032318791	0.6195327	0.0302429	0.5288041	0.7102613
20	42143096	cg02611863	L3MBTL:alt-TSS-DMR	0.6337061	0.032655164	0.6010509	0.0220061	0.5350326	0.6670692
20	42143174	cg15330298	L3MBTL:alt-TSS-DMR	0.6752344	0.044238436	0.630996	0.0257285	0.5538105	0.7081815
20	42143211	cg20252111	L3MBTL:alt-TSS-DMR	0.5645233	0.004054518	0.5604688	0.0157086	0.5133431	0.6075945
20	42143399	cg15302378	L3MBTL:alt-TSS-DMR	0.6928797	0.031301091	0.6615786	0.021766	0.5962806	0.7268766
20	42143489	cg09541000	L3MBTL:alt-TSS-DMR	0.6745273	0.041165755	0.6333615	0.0174246	0.5810877	0.6856354
20	42143502	cg02472486	L3MBTL:alt-TSS-DMR	0.5831913	0.0277043	0.555487	0.022685	0.4874319	0.6235421
20	57414039	cg04132853	NESP-DMR	0.6220468	-0.017099955	0.6391468	0.0209355	0.5763401	0.7019534
20	57414059	cg25090051	GNAS-NESP-DMR	0.6014206	-0.014919918	0.6163405	0.0145724	0.5726235	0.6600576
20	57414162	cg00732970	GNAS-NESP-DMR	0.5380618	-0.004490345	0.5425521	0.0161471	0.4941107	0.5909936
20	57414217	cg17696847	GNAS-NESP-DMR	0.5691358	-0.0108309	0.5799667	0.0162091	0.5313394	0.628594
20	57414274	cg23732978	GNAS-NESP-DMR	0.6324916	-0.026788164	0.6592798	0.0666336	0.4593791	0.8591805
20	57414351	cg20019489	GNAS-NESP-DMR	0.7351562	-0.000619573	0.7357758	0.0213751	0.6716505	0.7999011
20	57414407	cg02274728	GNAS-NESP-DMR	0.6202478	0.003224127	0.6170237	0.0199318	0.5572283	0.6768191
20	57414529	cg12054318	GNAS-NESP-DMR	0.5959214	0.015654782	0.5802666	0.0176113	0.5274327	0.6331005
20	57414539	cg25268451	GNAS-NESP-DMR	0.6398569	0.019158209	0.6206987	0.0155887	0.5739325	0.6674649
20	57414578	cg11996914	GNAS-NESP-DMR	0.5486853	-0.014902973	0.5635883	0.019157	0.5061173	0.6210593
20	57414596	cg21330323	GNAS-NESP-DMR	0.5082479	-0.014722891	0.5229708	0.0181745	0.4684474	0.5774942
20	57414884	cg06163629	GNAS-NESP-DMR	0.523497	-0.008833991	0.532331	0.0233533	0.4622712	0.6023908
20	57414960	cg14597908	GNAS-NESP-DMR	0.5542287	0.005518255	0.5487104	0.0106232	0.5168409	0.58058
20	57415144	cg00765653	GNAS-NESP-DMR	0.5918965	-0.010777182	0.6026737	0.0198849	0.5430191	0.6623282
20	57415177	cg14728235	GNAS-NESP-DMR	0.5740691	-0.020377227	0.5944463	0.012627	0.5565652	0.6323274
20	57415377	cg05558390	GNAS-NESP-DMR	0.394962	-0.000814082	0.3957761	0.0254352	0.3194704	0.4720818
20	57415697	cg19296354	GNAS-NESP-DMR	0.5755934	0.007760791	0.5678326	0.0172849	0.5159778	0.6196875
20	57416221	cg24975842	GNAS-NESP-DMR	0.5099819	-0.010977091	0.520959	0.0196585	0.4619834	0.5799346
20	57416506	cg18619398	GNAS-NESP-DMR	0.514245	-0.005470291	0.5197153	0.0163223	0.4707484	0.5686822
20	57416888	cg01355739	GNAS-NESP-DMR	0.6309069	0.016857064	0.6140498	0.016698	0.563956	0.6641437
20	57417152	cg00952145	GNAS-NESP-DMR	0.4709794	-0.002663082	0.4736425	0.0135378	0.4330292	0.5142558

20	57417233	cg20582984	GNAS-NESP-DMR	0.6930838	0.013305109	0.6797787	0.0109761	0.6468503	0.7127071
20	57418015	cg18949315	GNAS-NESP-DMR	0.5694832	-0.003444473	0.5729277	0.0178184	0.5194726	0.6263828
20	57425979	cg18935491	GNAS-AS1:TSS-DMR	0.5677596	0.023236118	0.5445235	0.0208383	0.4820084	0.6070385
20	57425986	cg24203465	GNAS-AS1:TSS-DMR	0.5760812	0.035511264	0.5405699	0.017714	0.4874279	0.5937119
20	57425994	cg26102503	GNAS-AS1:TSS-DMR	0.5608935	0.039294727	0.5215988	0.0232176	0.4519461	0.5912515
20	57426131	cg03344105	GNAS-AS1:TSS-DMR	0.6029852	0.062431045	0.5405542	0.0200566	0.4803845	0.6007238
20	57426138	cg14660776	GNAS-AS1:TSS-DMR	0.5330718	0.054503309	0.4785685	0.019612	0.4197326	0.5374044
20	57426215	cg19592829	GNAS-AS1:TSS-DMR	0.5678697	0.036624682	0.531245	0.0170925	0.4799675	0.5825226
20	57426240	cg20401058	GNAS-AS1:TSS-DMR	0.5570726	0.037139609	0.519933	0.0310919	0.4266573	0.6132087
20	57426264	cg09123158	GNAS-AS1:TSS-DMR	0.6918337	0.017300082	0.6745336	0.0182592	0.6197561	0.7293111
20	57426274	cg19140375	GNAS-AS1:TSS-DMR	0.5510428	0.034573236	0.5164696	0.0203473	0.4554277	0.5775114
20	57426322	cg11669839	GNAS-AS1:TSS-DMR	0.5115552	0.036822491	0.4747327	0.0241886	0.4021668	0.5472986
20	57426368	cg17841572	GNAS-AS1:TSS-DMR	0.5462556	0.042472064	0.5037835	0.0192968	0.4458931	0.561674
20	57426374	cg25229306	GNAS-AS1:TSS-DMR	0.5887658	0.033618927	0.5551469	0.0156646	0.508153	0.6021407
20	57426383	cg14482474	GNAS-AS1:TSS-DMR	0.5962818	0.0346348	0.561647	0.0123067	0.5247268	0.5985672
20	57426391	cg08166863	GNAS-AS1:TSS-DMR	0.5518227	0.0170839	0.5347388	0.0170174	0.4836866	0.585791
20	57426395	cg11285807	GNAS-AS1:TSS-DMR	0.5045905	0.028011573	0.4765789	0.0183248	0.4216044	0.5315534
20	57426420	cg06200857	GNAS-AS1:TSS-DMR	0.612471	0.032483673	0.5799873	0.0132432	0.5402576	0.619717
20	57426425	cg08091561	GNAS-AS1:TSS-DMR	0.5594506	0.017577291	0.5418733	0.013591	0.5011004	0.5826462
20	57426538	cg22860367	GNAS-AS1:TSS-DMR	0.4893456	-0.003368827	0.4927144	0.0166637	0.4427233	0.5427056
20	57426545	cg07947033	GNAS-AS1:TSS-DMR	0.5283189	0.014536391	0.5137825	0.0242144	0.4411394	0.5864256
20	57426570	cg06693667	GNAS-AS1:TSS-DMR	0.560668	0.019447418	0.5412206	0.0164664	0.4918214	0.5906198
20	57426580	cg15631127	GNAS-AS1:TSS-DMR	0.5552654	0.0221692	0.5330962	0.0197468	0.4738558	0.5923366
20	57426626	cg23484981	GNAS-AS1:TSS-DMR	0.6010209	0.1137288	0.4872921	0.0236114	0.4164578	0.5581264
20	57426743	cg04677683	GNAS-AS1:TSS-DMR	0.5681556	0.049936855	0.5182187	0.0199986	0.458223	0.5782145
20	57426749	cg15160445	GNAS-AS1:TSS-DMR	0.6188375	0.047019455	0.571818	0.0139327	0.5300199	0.6136162
20	57426757	cg25326570	GNAS-AS1:TSS-DMR	0.5853058	0.036823482	0.5484823	0.0148309	0.5039896	0.5929751
20	57426759	cg23249369	GNAS-AS1:TSS-DMR	0.5935774	0.040784027	0.5527934	0.0276927	0.4697154	0.6358714
20	57426789	cg01715551	GNAS-AS1:TSS-DMR	0.5199963	0.021825136	0.4981712	0.0171368	0.4467607	0.5495816
20	57426801	cg14176797	GNAS-AS1:TSS-DMR	0.5481766	0.0224941	0.5256825	0.019069	0.4684755	0.5828895
20	57426835	cg04457481	GNAS-AS1:TSS-DMR	0.5573694	0.033785545	0.5235839	0.0275063	0.441065	0.6061027
20	57426858	cg27262796	GNAS-AS1:TSS-DMR	0.5389401	0.0018274	0.5371127	0.020024	0.4770407	0.5971847
20	57426931	cg21938532	GNAS-AS1:TSS-DMR	0.5440785	0.014317345	0.5297612	0.0231601	0.4602808	0.5992415
20	57426935	cg03606258	GNAS-AS1:TSS-DMR	0.5588157	0.012270345	0.5465454	0.0180597	0.4923661	0.6007246
20	57426950	cg22989942	GNAS-AS1:TSS-DMR	0.5844365	0.032986773	0.5514497	0.0235134	0.4809094	0.6219901
20	57426979	cg18224653	GNAS-AS1:TSS-DMR	0.5082124	0.011109364	0.497103	0.0184459	0.4417653	0.5524408
20	57427010	cg14309385	GNAS-AS1:TSS-DMR	0.5459148	0.019345227	0.5265696	0.0168271	0.4760882	0.577051
20	57427017	cg05309239	GNAS-AS1:TSS-DMR	0.4947415	0.004083927	0.4906576	0.0206263	0.4287788	0.5525364
20	57427030	cg12573482	GNAS-AS1:TSS-DMR	0.5067054	0.010612991	0.4960924	0.0119946	0.4601086	0.5320762
20	57427046	cg01017090	GNAS-AS1:TSS-DMR	0.4550138	-0.014338891	0.4693527	0.0200538	0.4091913	0.5295141
20	57427103	cg06324048	GNAS-AS1:TSS-DMR	0.5829386	0.004199355	0.5787392	0.0176495	0.5257907	0.6316878
20	57427117	cg13728472	GNAS-AS1:TSS-DMR	0.4339668	0.011961545	0.4220053	0.0202422	0.3612786	0.4827319
20	57427146	cg24617313	GNAS-AS1:TSS-DMR	0.5140319	0.041925309	0.4721066	0.0200644	0.4119134	0.5322998
20	57427170	cg03908391	GNAS-AS1:TSS-DMR	0.550395	-0.051825591	0.6022206	0.0333721	0.5021044	0.7023368
20	57427173	cg22290117	GNAS-AS1:TSS-DMR	0.5810763	-0.0264231	0.6074994	0.0178665	0.5538998	0.661099
20	57427210	cg26496204	GNAS-AS1:TSS-DMR	0.521355	-0.025874173	0.5472292	0.0171498	0.4957798	0.5986786
20	57427237	cg16833551	GNAS-AS1:TSS-DMR	0.6595154	-0.025777818	0.6852932	0.0160735	0.6370727	0.7335137
20	57427274	cg03010274	GNAS-AS1:TSS-DMR	0.4624781	-0.018279909	0.480758	0.0170841	0.4295056	0.5320105
20	57427412	cg25652859	GNAS-AS1:TSS-DMR	0.3752471	-0.027997564	0.4032447	0.0200931	0.3429652	0.4635241
20	57427426	cg12321149	GNAS-AS1:TSS-DMR	0.3756154	-0.032645436	0.4082608	0.0267248	0.3280863	0.4884353
20	57427443	cg06065549	GNAS-AS1:TSS-DMR	0.4389316	-0.0233979	0.4623295	0.017712	0.4091935	0.5154655
20	57427472	cg07105596	GNAS-AS1:TSS-DMR	0.3215531	-0.074188973	0.3957421	0.0359071	0.2880207	0.5034635
20	57427483	cg27304369	GNAS-AS1:TSS-DMR	0.3796889	-0.055559118	0.435248	0.0398202	0.3157874	0.5547087
20	57427493	cg26811638	GNAS-AS1:TSS-DMR	0.3558612	-0.037145155	0.3930064	0.0222807	0.3261643	0.4598484
20	57427495	cg26534489	GNAS-AS1:TSS-DMR	0.4235607	-0.000494255	0.424055	0.0200905	0.3637834	0.4843265
20	57427503	cg08587534	GNAS-AS1:TSS-DMR	0.4883096	-0.016300291	0.5046099	0.0285445	0.4189764	0.5902434
20	57427556	cg14564778	GNAS-AS1:TSS-DMR	0.3790022	-0.077779827	0.456782	0.0268393	0.3762642	0.5372999
20	57427642	cg01817393	GNAS-AS1:TSS-DMR	0.3775123	-0.047340218	0.4248525	0.0475492	0.2822048	0.5675002
20	57427713	cg04257105	GNAS-AS1:TSS-DMR	0.3610922	-0.075032855	0.4361251	0.0206485	0.3741794	0.4980707
20	57427730	cg20528838	GNAS-AS1:TSS-DMR	0.25973	-0.056579364	0.3163094	0.0249212	0.2415456	0.3910731
20	57427738	cg27661264	GNAS-AS1:TSS-DMR	0.3228914	-0.051813509	0.3747049	0.0238172	0.3032533	0.4461565
20	57427762	cg19589727	GNAS-AS1:TSS-DMR	0.3278497	-0.053567282	0.381417	0.0218966	0.3157273	0.4471067
20	57427821	cg10302550	GNAS-AS1:TSS-DMR	0.344601	-0.087094418	0.4316954	0.0265327	0.3520974	0.5112934
20	57427830	cg17414107	GNAS-AS1:TSS-DMR	0.4317812	-0.082564991	0.5143462	0.0285998	0.4285468	0.6001456
20	57427942	cg00943909	GNAS-AS1:TSS-DMR	0.3374061	-0.092556836	0.4299629	0.0311181	0.3366086	0.5233173
20	57427973	cg19640589	GNAS-AS1:TSS-DMR	0.3755421	-0.100818536	0.4763606	0.0331892	0.376793	0.5759283
20	57428032	cg24346429	GNAS-AS1:TSS-DMR	0.495022	0.012956473	0.4820655	0.0340421	0.3799393	0.5841917
20	57429277	cg21988465	GNAS XL:Ex1-DMR	0.6453789	0.066976027	0.5784029	0.0369792	0.4674654	0.6893404
20	57429858	cg07284407	GNAS XL:Ex1-DMR	0.6260459	0.055417545	0.5706284	0.0137138	0.5294868	0.6117699
20	57430313	cg21625881	GNAS XL:Ex1-DMR	0.5963187	0.025862764	0.5704559	0.0144152	0.5272102	0.6137017
20	57430663	cg14203179	GNAS XL:Ex1-DMR	0.5882595	0.063621518	0.524638	0.0301041	0.4343258	0.6149502
20	57431202	cg09437522	GNAS XL:Ex1-DMR	0.5454521	0.0652881	0.480164	0.0192396	0.4224452	0.5378828
20	57431303	cg10468484	GNAS XL:Ex1-DMR	0.5557706	0.046198073	0.5095725	0.0283902	0.4244019	0.5947432
20	57463265	cg22741626	GNAS A/B:TSS-DMR	0.7470357	0.037741945	0.7092938	0.0131837	0.6697428	0.7488447
20	57463270	cg18997188	GNAS A/B:TSS-DMR	0.7048068	0.045261082	0.6595457	0.0153469	0.6135052	0.7055863
20	57463325	cg20213508	GNAS A/B:TSS-DMR	0.7395158	0.023229782	0.716286	0.0248937	0.641605	0.7909671
20	57463330	cg26791489	GNAS A/B:TSS-DMR	0.6538739	0.033849145	0.6200248	0.022667	0.5520239	0.6880257
20	57463355	cg04779428	GNAS A/B:TSS-DMR	0.6922948	0.028612627	0.6636822	0.0176216	0.6108175	0.7165468
20	57463357	cg04019914	GNAS A/B:TSS-DMR	0.7523901	0.0480161	0.704374	0.0226262	0.6364955	0.7722525

20	57463397	cg11357538	GNAS A/B:TSS-DMR	0.5865278	0.069119909	0.5174079	0.016622	0.4675418	0.567274
20	57463455	cg20008140	GNAS A/B:TSS-DMR	0.6405765	0.077678527	0.562898	0.0297253	0.473722	0.652074
20	57463503	cg11480267	GNAS A/B:TSS-DMR	0.579989	0.0812745	0.4987145	0.0273571	0.4166432	0.5807858
20	57463527	cg10011623	GNAS A/B:TSS-DMR	0.5806833	0.106024582	0.4746587	0.0289443	0.3878257	0.5614917
20	57463530	cg01748573	GNAS A/B:TSS-DMR	0.6316016	0.111875973	0.5197256	0.028996	0.4327377	0.6067135
20	57463572	cg17334845	GNAS A/B:TSS-DMR	0.5488331	0.082302482	0.4665306	0.0186651	0.4105355	0.5225258
20	57463615	cg26767990	GNAS A/B:TSS-DMR	0.4957165	0.068278473	0.427438	0.0260822	0.3491914	0.5056847
20	57463653	cg17652507	GNAS A/B:TSS-DMR	0.5917841	0.124533218	0.4672509	0.0356124	0.3604135	0.5740882
20	57463658	cg22407822	GNAS A/B:TSS-DMR	0.6257529	0.128907291	0.4968456	0.0308636	0.4042547	0.5894365
20	57463711	cg07341934	GNAS A/B:TSS-DMR	0.6458454	0.094467664	0.5513777	0.0216416	0.4864528	0.6163026
20	57463713	cg02107718	GNAS A/B:TSS-DMR	0.611078	0.106862709	0.5042153	0.0220985	0.4379199	0.5705107
20	57463725	cg23496597	GNAS A/B:TSS-DMR	0.6149983	0.107239318	0.507759	0.033727	0.406578	0.60894
20	57463763	cg25308079	GNAS A/B:TSS-DMR	0.5716346	0.054343282	0.5172913	0.0165751	0.4675662	0.5670165
20	57463767	cg03014008	GNAS A/B:TSS-DMR	0.5691991	0.114401655	0.4547974	0.0332046	0.3551837	0.5544112
20	57463775	cg09772382	GNAS A/B:TSS-DMR	0.6088333	0.060551882	0.5482814	0.0210562	0.4851128	0.6114501
20	57463783	cg15222215	GNAS A/B:TSS-DMR	0.6031545	0.099462055	0.5036924	0.0269809	0.4227499	0.584635
20	57463787	cg14263118	GNAS A/B:TSS-DMR	0.6309674	0.083740445	0.547227	0.0274748	0.4648025	0.6296514
20	57463900	cg11244758	GNAS A/B:TSS-DMR	0.5590405	0.075731155	0.4833093	0.0181941	0.428727	0.5378917
20	57463903	cg18160880	GNAS A/B:TSS-DMR	0.6972638	0.106384055	0.5908797	0.0157392	0.5436623	0.6380972
20	57463906	cg05926269	GNAS A/B:TSS-DMR	0.7204542	0.103926782	0.6165274	0.0204813	0.5550835	0.6779713
20	57463925	cg03821543	GNAS A/B:TSS-DMR	0.5773746	0.102246082	0.4751285	0.0315248	0.3805542	0.5697028
20	57463974	cg01538522	GNAS A/B:TSS-DMR	0.4994116	0.075787373	0.4236242	0.0177339	0.3704226	0.4768258
20	57463984	cg00267746	GNAS A/B:TSS-DMR	0.4853348	0.068265318	0.4170695	0.0244197	0.3438103	0.4903286
20	57463991	cg09885502	GNAS A/B:TSS-DMR	0.6479617	0.118586727	0.529375	0.1538478	0.0678315	0.9909185
20	57464000	cg03837903	GNAS A/B:TSS-DMR	0.6361756	0.081965764	0.5542098	0.0224838	0.4867584	0.6216612
20	57464002	cg23159236	GNAS A/B:TSS-DMR	0.4338087	0.065954191	0.3678545	0.0292869	0.2799939	0.4557151
20	57464129	cg22798925	GNAS A/B:TSS-DMR	0.5854895	0.089198636	0.4962909	0.0247217	0.4221257	0.570456
20	57464571	cg20126878	GNAS A/B:TSS-DMR	0.57845	0.088301227	0.4901488	0.0181651	0.4356536	0.544644
20	57464742	cg27027803	GNAS A/B:TSS-DMR	0.6568602	0.108579473	0.5482807	0.0145132	0.504741	0.5918204
20	57464970	cg08997444	GNAS A/B:TSS-DMR	0.6925464	0.085562755	0.6069836	0.0181755	0.552457	0.6615103
20	57464973	cg22639787	GNAS A/B:TSS-DMR	0.7530596	0.100876364	0.6521832	0.0189655	0.5952869	0.7090796
20	57465123	cg05960039	GNAS A/B:TSS-DMR	0.4109491	0.0600113	0.3509378	0.0179682	0.2970331	0.4048425
20	57465125	cg09604333	GNAS A/B:TSS-DMR	0.4970074	0.0586078	0.4383996	0.0227285	0.370214	0.5065852
20	57465132	cg06047881	GNAS A/B:TSS-DMR	0.5090633	0.075973336	0.43309	0.0226619	0.3651042	0.5010757
20	57465139	cg20018057	GNAS A/B:TSS-DMR	0.5172144	0.075095827	0.4421186	0.0169548	0.391254	0.4929831
20	57465175	cg10748817	GNAS A/B:TSS-DMR	0.5876021	0.080959545	0.5066426	0.0322274	0.4099602	0.6033249
21	40757691	cg00606841	WRB:alt-TSS-DMR	0.5942925	-0.007088136	0.6013806	0.0249619	0.5264949	0.6762663
21	40757750	cg22858667	WRB:alt-TSS-DMR	0.5902305	-0.002822236	0.5930527	0.0159479	0.5452091	0.6408963
21	40757899	cg26710963	WRB:alt-TSS-DMR	0.5761603	0.007457482	0.5687028	0.0136069	0.527882	0.6095236
21	40758208	cg09916765	WRB:alt-TSS-DMR	0.4400557	-0.025475236	0.4655309	0.0158322	0.4180342	0.5130277
22	42077939	cg06781532	SNU13:alt-TSS-DMR	0.6637015	0.008965291	0.6547362	0.0210985	0.5914408	0.7180317
22	42078217	cg18152773	SNU13:alt-TSS-DMR	0.5142947	0.0171296	0.4971651	0.0173987	0.4449691	0.5493611
22	42078330	cg05871614	SNU13:alt-TSS-DMR	0.5680677	0.026000264	0.5420674	0.0168232	0.491598	0.5925369
22	42078365	cg22083753	SNU13:alt-TSS-DMR	0.5255094	0.015569064	0.5099403	0.0173654	0.457844	0.5620367
22	42078388	cg15284719	SNU13:alt-TSS-DMR	0.5150444	0.009306236	0.5057382	0.012883	0.467089	0.5443873
22	42078567	cg08686092	SNU13:alt-TSS-DMR	0.5314486	0.032277264	0.4991713	0.0211169	0.4358205	0.5625221
22	42078707	cg11677105	SNU13:alt-TSS-DMR	0.4597698	0.014998091	0.4447717	0.0396019	0.3259661	0.5635774

We calculated the average and standard deviation (SD) of β-value at each probe (CpG site) using leukocyte genomic DNA samples from 11 control subjects, and obtained the differences between the β-value of this patient and the average β-value of the control group (Δβ). The methylation pattern of each probe (CpG site) is interpreted as abnormal, when the |Δβ| is > 3 SD and > 0.05. When > 20% of probes within a DMR showed abnormal methylation levels, the DMR is considered aberrantly methylated.

In the column of "Average" of this patient, the β-values above AVG + >3 SD and < AVG −3 SD of the control subjects are written in red and blue, respectively.

In the column of "Δβ" of this patient, abnormal values are higlighted with different colors corresponding to those in figure 1B.

Table S3 The results of micosatellite analysis

Locus	Position	Mother	Patient	Father	Assesment
D14S608	14q12	205/213	205/209	209	Biparental
D14S588	14q23-24.1	114	114/122	114/122	Biparental or paternal heterodisomy
D14S617	14q32.12	139/165	165	165/169	Biparental or uniparental isodisomy
D14S1006	14q32.2	126/134	126/134	126	Biparental or maternal heterodisomy
D14S985	14q32.2	137	131/137	131/137	Biparental or paternal heterodisomy
D14S1010	14q32.33	144/148	144/148	144/148	Biparental or uniparental heterodisomy
D14S292	14q32.33	104/110	104/110	110	Biparental or maternal heterodisomy

The overall findings inidcate biparebntal originof the chromosome 14 homologs in this patient.

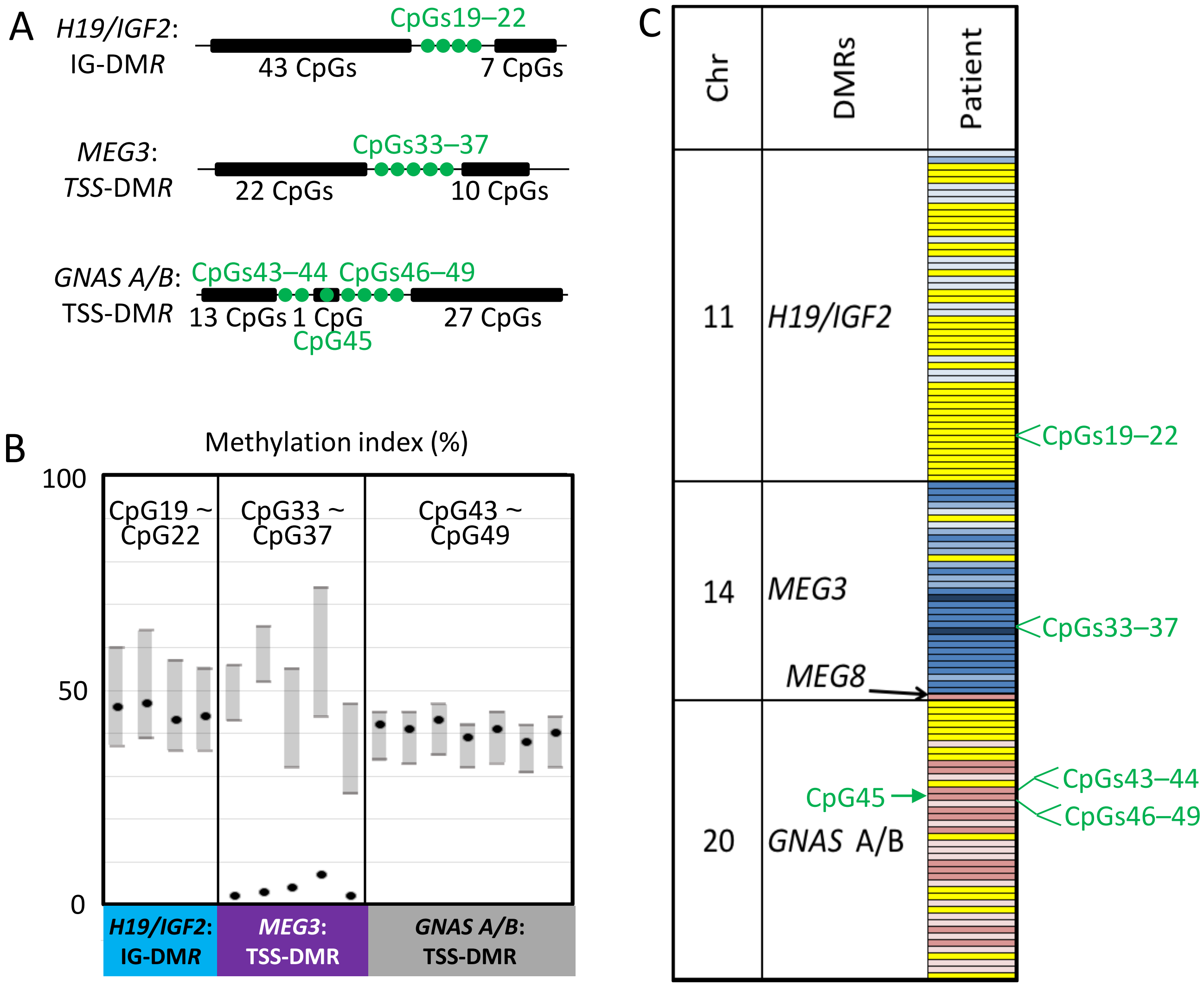

Figure S1 Methylation analyses of the *H19/IGF2*:IG-DMR, *MEG3*:TSS-DMR, and *GNAS A/B*:TSS-DMR, using leukocyte gDNA samples.

A. Schematic representation showing the relative positions of CpGs examined by pyrosequencing (green dots) and HumanMethylation450 BeadChip analysis (black bars).
B. The results of pyrosequencing analysis.
C. The results of HumanMethylation450 BeadChip analysis. The positions of CpGs examined by pyrosequencing are indicated. CpG45 has been examined by both analyses.

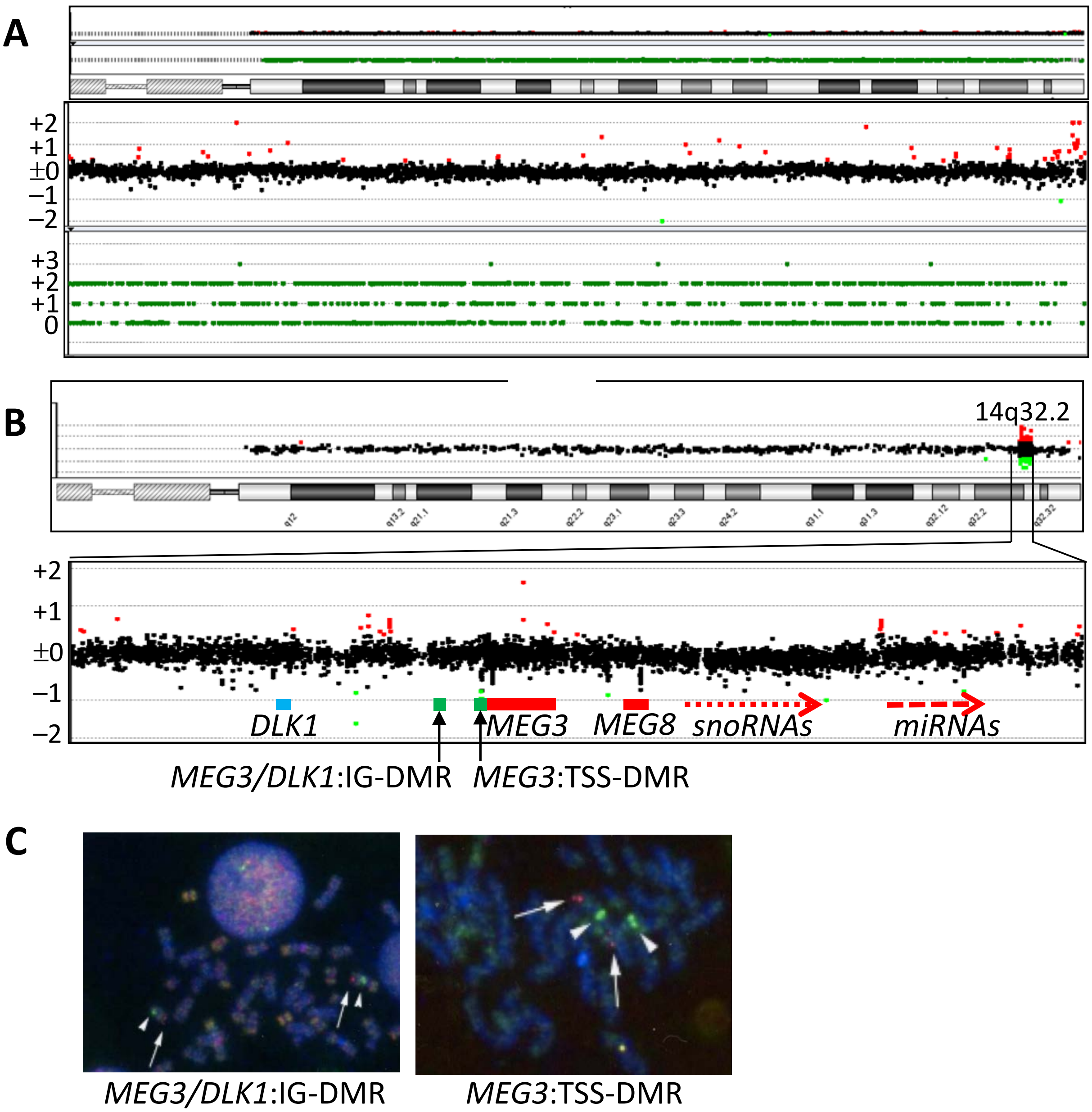

Figure S2 Lack of UPD(14)mat and microdeletion in this patient.

A. The finding of CGH+SNP microarray.

B. The finding of a custom-build oligo-microarray for 14q32.2–q32.3.

C. FISH results for the *MEG3/DLK1*:IG-DMR and the *MEG3*:TSS-DMR. The red signals (arrows) have been detected by probes for the *MEG3/DLK1*:IG-DMR or *MEG3*:TSS-DMR, and the green signals (arrowheads) have been identified by an RP11-566I2 probe for 14q12 used as an internal control.

Case description in the original papers				This case	Pt.1	Pt.3	18	21	23	29	31	32	34	35	36	37	1	13	BWS-102	BWS-s001	BWS-s011	BWS-s023	BWS-s062	BWS-s113	BWS-s015	BWS-s064	OGS 1000	OGS 1052
Reference					1	1	2	2	2	2	2	2	2	2	2	2	3	3	4	4	4	4	4	4	4	4	5	5
Clinical phenotype				SRS	PWS	SRS	BWS	SRS	BWS	BWS	SRS	BWS	SRS	SRS	PHP1b	SRS-like	SRS	SRS	BWS	BWS	BWS	BWS	BWS	BWS	BWS	BWS	BWS	BWS
Disease-causing epimutated DMR				*MEG3*	*MEG3/DLK1 MEG3*	*MEG3/DLK1 MEG3*	*KCNQ1OT1*	*H19/IGF2*	*KCNQ1OT1*	*KCNQ1OT1*	*KCNQ1OT1*	*KCNQ1OT1*	*KCNQ1OT1*	Uncertain	*GNAS A/B*	*H19/IGF2 KCNQ1OT1*	*MEG3/DLK 1 MEG3*	*MEG3/DLK 1 MEG3*	*KCNQ1OT1*	*KCNQ1OT1*	*KCNQ1OT1*	*KCNQ1OT1*	*KCNQ1OT1*	*KCNQ1OT1*	*H19/IGF2*	*H19/IGF2*	*KCNQ1OT1*	*KCNQ1OT1*
Methylation analysis methods					Pyrosequencing Illumina 450K BeadChip		Illumina 450K BeadChip										ASMM RTQ-PCR MS-MLPA		MALDI-TOF MS Pyrosequencing								A custom Illumina Veracode	
DMR*	Chr	Methylated allele*	Character of DMR*																									
PPIE L:Ex1-DMR	1	Maternal	O gDMR																									
DIRAS3 :TSS-DMR	1	Maternal	O gDMR																									
DIRAS3 :Ex2-DMR	1	Maternal	O gDMR																									
ZDBF2/GPR1 :IG-DMR	2	Paternal	Sp gDMR-Sec DMR																									
NAP1L5 :TSS-DMR	4	Maternal	O gDMR																									
FAM50B :TSS-DMR	6	Maternal	O gDMR																									
PLAGL1 :alt-TSS-DMR	6	Maternal	O gDMR																									
IGF2R :Int2-DMR	6	Maternal	O gDMR																									
WDR27 :Int13-DMR	6	Maternal	O gDMR																									
GRB10 :alt-TSS-DMR	7	Maternal	O gDMR																									
PEG10 :TSS-DMR	7	Maternal	O gDMR																									
MEST :alt-TSS-DMR	7	Maternal	O gDMR																									
HTR5A :TSS-DMR	7	Maternal	O gDMR																									
ERLIN2 :Int6-DMR	8	Maternal	O gDMR																									
PEG13 :TSS-DMR	8	Maternal	O gDMR																									
INPP5 F:Int2-DMR	10	Maternal	O gDMR																									
H19/IGF2 :IG-DMR	11	Paternal	Sp gDMR																									
IGF2 :Ex9-DMR	11	Paternal	Sec DMR																									
IGF2 :alt-TSS-DMR	11	Paternal	Sp gDMR																									
KCNQ1OT1 :TSS-DMR	11	Maternal	O gDMR																									
RB1 :Int2-DMR	13	Maternal	O gDMR																									
MEG3/DLK1 :IG-DMR	14	Paternal	Sp gDMR																									
MEG3 :TSS-DMR	14	Paternal	Sec DMR																									
MEG8 :Int2-DMR	14	Maternal	Sec DMR																									
MKRN3 :TSS-DMR	15	Maternal	O gDMR-SecDMR																									
MAGEL2 :TSS-DMR	15	Maternal	Sec DMR																									
NDN :TSS-DMR	15	Maternal	Sec DMR																									
SNRPN :alt-TSS-DMR	15	Maternal	Sec DMR																									
SNURF :TSS-DMR	15	Maternal	O gDMR																									
IGF1R :Int2-DMR	15	Maternal	O gDMR																									
ZNF597 :3' DMR	16	Maternal	O gDMR																									
ZNF597 :TSS-DMR	16	Paternal	Sec DMR																									
ZNF331 :alt-TSS-DMR1	19	Maternal	O gDMR																									
ZNF331 :alt-TSS-DMR2	19	Maternal	O gDMR																									
PEG3 :TSS-DMR	19	Maternal	O gDMR																									
MCTS2P :TSS-DMR	20	Maternal	O gDMR																									
NNAT :TSS-DMR	20	Maternal	O gDMR																									
L3MBTL1 :alt-TSS-DMR	20	Maternal	O gDMR																									
GNAS-NESP :TSS-DMR	20	Paternal	Sec DMR																									
GNAS-AS1 :TSS-DMR	20	Maternal	O gDMR																									
GNAS-XL: Ex1-DMR	20	Maternal	O gDMR																									
GNAS A/B :TSS-DMR	20	Maternal	Sec DMR																									
WRB :alt-TSS-DMR	21	Maternal	O gDMR																									
SNU13 :alt-TSS-DMR	22	Maternal	O gDMR																									

Figure S3 Representative data in patients with both hypomethylation-type and hypermethylation-type of MLID in the absence of a mutation in causative or candidate genes for MLID.
* We have adopted Standardized nomenclature for imprinted loci/differentially methylated region in web site of European Network for Human Congenital Imprinting Disorders (http://www.imprinting-disorders.eu/).
The blue, red, and green boxes represent DMRs assessed to be hypomethylated, hypermethylated, and normally methylated, respectively, by the methods employed in each case; the gray boxes indicate non-examined DMRs.
Abbreviations. O gDNA: Oocyte germline DMR, Sp gDMR: Sperm germline DMR, Sec-DMR: Secondary DMR, SRS: Silver-Russell syndrome, BWS: Beckwith-Wiedemann syndrome, PHP1b: Pseudohypoparathyroidism 1b, TNDM: Transient neonatal diabetes mellitus, ASMM RTQ-PCR: TaqMan allele-specific methylated multiplex real-time quantitative PCR, and MALDI-TOF MS: Matrix Assisted Laser Desorption Ionization-Time of Flight Mass Spectrometry.

References

1. Kagami M, Matsubara K, Nakabayashi K, Nakamura A, Sano S, Okamura K, et al. Genome-wide multilocus imprinting disturbance analysis in Temple syndrome and Kagami-Ogata syndrome. Genet Med. 2017;19:476–82.
2. Bens S, Kolarova J, Beygo J, Buiting K, Caliebe A, Eggermann T, et al. Phenotypic spectrum and extent of DNA methylation defects associated with multilocus imprinting disturbances. Epigenomics. 2016;8:801-16.
3. Geoffron S, Abi Habib W, Chantot-Bastaraud S, Dubern B, Steunou V, Azzi S, Afenjar A, et al. .Chromosome 14q32.2 Imprinted Region Disruption as an Alternative Molecular Diagnosis of Silver-Russell Syndrome. J Clin Endocrinol Metab. 2018;103:2436-46.
4. Maeda T, Higashimoto K, Jozaki K, Yatsuki H, Nakabayashi K, Makita Y, et al. Comprehensive and quantitative multilocus methylation analysis reveals the susceptibility of specific imprinted differentially methylated regions to aberrant methylation in Beckwith-Wiedemann syndrome with epimutations. Genet Med. 2014;16:903-12.
5. Court F, Martin-Trujillo A, Romanelli V, Garin I, Iglesias-Platas I, Salafsky I, et al. Genome-wide allelic methylation analysis reveals disease-specific susceptibility to multiple methylation defects in imprinting syndromes. Hum Mutat. 2013;34:595-602.
